# Immunodominance and functional alterations of tumor‐associated antigen‐specific CD8^+^ T‐cell responses in hepatocellular carcinoma

**DOI:** 10.1002/hep.26731

**Published:** 2014-02-20

**Authors:** Tobias Flecken, Nathalie Schmidt, Sandra Hild, Emma Gostick, Oliver Drognitz, Robert Zeiser, Peter Schemmer, Helge Bruns, Thomas Eiermann, David A. Price, Hubert E. Blum, Christoph Neumann‐Haefelin, Robert Thimme

**Affiliations:** ^1^ Department of Internal Medicine IIUniversity Hospital FreiburgFreiburgGermany; ^2^ Spemann Graduate School of Biology and Medicine (SGBM)Albert‐Ludwigs‐UniversityFreiburgGermany; ^3^ Faculty of BiologyAlbert‐Ludwigs‐UniversityFreiburgGermany; ^4^ Institute of Infection and ImmunityCardiff University School of MedicineCardiffUK; ^5^ Department of SurgeryUniversity Hospital FreiburgFreiburgGermany; ^6^ Department of Internal Medicine IUniversity Hospital FreiburgFreiburgGermany; ^7^ Department of General and Transplant SurgeryRuprecht‐Karls UniversityHeidelbergGermany; ^8^ Department of Transfusion MedicineUniversity Hospital Hamburg‐EppendorfHamburgGermany

## Abstract

Hepatocellular carcinoma (HCC) is the fifth most common malignancy worldwide with a poor prognosis and limited therapeutic options. To aid the development of novel immunological interventions, we studied the breadth, frequency, and tumor‐infiltration of naturally occurring CD8^+^ T‐cell responses targeting several tumor‐associated antigens (TAA). We used overlapping peptides spanning the entire alpha‐fetoprotein (AFP), glypican‐3 (GPC‐3), melanoma‐associated gene‐A1 (MAGE‐A1) and New York‐esophageal squamous cell carcinoma‐1 (NY‐ESO‐1) proteins and major‐histocompatibility‐complex‐class‐I‐tetramers specific for epitopes of MAGE‐A1 and NY‐ESO‐1 to analyze TAA‐specific CD8^+^ T‐cell responses in a large cohort of HCC patients. After nonspecific expansion *in vitro*, we detected interferon‐γ (IFN‐γ)‐producing CD8^+^ T cells specific for all four TAA in the periphery as well as in liver and tumor tissue. These CD8^+^ T‐cell responses displayed clear immunodominance patterns within each TAA, but no consistent hierarchy was observed between different TAA. Importantly, the response breadth was highest in early‐stage HCC and associated with patient survival. After antigen‐specific expansion, TAA‐specific CD8^+^ T cells were detectable by tetramer staining but impaired in their ability to produce IFN‐γ. Furthermore, regulatory T cells (T_reg_) were increased in HCC lesions. Depletion of T_reg_ from cultures improved TAA‐specific CD8^+^ T‐cell proliferation but did not restore IFN‐γ‐production. *Conclusion*: Naturally occurring TAA‐specific CD8^+^ T‐cell responses are present in patients with HCC and therefore constitute part of the normal T‐cell repertoire. Moreover, the presence of these responses correlates with patient survival. However, the observation of impaired IFN‐γ production suggests that the efficacy of such responses is functionally limited. These findings support the development of strategies that aim to enhance the total TAA‐specific CD8^+^ T‐cell response by therapeutic boosting and/or specificity diversification. However, further research will be required to help unlock the full potential of TAA‐specific CD8^+^ T‐cell responses. (Hepatology 2014;59:1415‐1426)

**Abbreviations**
AFP: α‐fetoprotein
BCLC: Barcelona Clinic Liver Cancer
CMV: cytomegalovirus
DMSO: dimethyl sulfoxide
EBV: Epstein‐Barr virus
EDTA: ethylenediaminetetraacetate
flu, influenza virus; GPC‐3: glypican‐3
HBV: hepatitis B virus
HCC: hepatocellular carcinoma
HCV: hepatitis C virus
HLA: human leukocyte antigen
IFN‐γ: interferon‐γ
IHL: intrahepatic lymphocytes
IL: interleukin
mAb: monoclonal antibody
MAGE‐A1: melanoma‐associated gene‐A1
NASH: nonalcoholic steatohepatitis
NY‐ESO‐1: New York‐esophageal squamous cell carcinoma‐1
PBMC: peripheral blood mononuclear cells
PE: phycoerythrin
PD‐1: programmed death‐1
PD‐L1: programmed death‐ligand 1
PFS: progression‐free survival
RFTA: radiofrequency thermal ablation
RPMI: Roswell Park Memorial Institute
TAA: tumor‐associated antigen
TACE: transarterial chemoembolization
TCR: T‐cell receptor
TIL: tumor‐infiltrating lymphocytes
Tim‐3: T‐cell immunoglobulin and mucin‐3
Treg: regulatory T cell

Hepatocellular carcinoma (HCC) is one of the most common cancers worldwide. Despite its growing incidence, however, therapeutic options remain limited. Consequently, HCC patients suffer from a high mortality rate.^1^ New therapies for HCC are therefore urgently required. Immunotherapy is a promising approach for the treatment of HCC.^2^ The rationale for immunological intervention is based on the presence of high numbers of tumor‐infiltrating T cells in HCC tissue,^3^ the correlation between the density of lymphocytic infiltrates in HCC lesions and prognosis,^4^ and, most importantly, the finding that adoptive immunotherapy with interleukin (IL)−2/anti‐CD3‐stimulated autologous lymphocytes lowers postsurgical recurrence rates in humans.^5^ The central effectors in this scenario are CD8^+^ T cells that recognize tumor‐associated antigens (TAA) and kill tumor cells. TAA comprise a range of self‐derived proteins rendered immunogenic in tumors either by mutation or aberrant expression.^6^ A variety of different TAA can spontaneously induce CD8^+^ T‐cell responses in HCC patients; these include α‐fetoprotein (AFP), glypican‐3 (GPC‐3), melanoma‐associated gene‐A1 (MAGE‐A1), and New York‐esophageal squamous cell carcinoma‐1 (NY‐ESO‐1).^7^ In addition, TAA‐specific responses can also be boosted *in vivo* in HCC patients by dendritic cell‐based vaccination with tumor lysate.^11^

The identification of TAA that are frequently recognized by CD8^+^ T cells in HCC patients could provide important insights into the choice of appropriate targets for immunotherapy. However, most previous studies focused on single TAA, thus precluding within‐patient comparisons. Indeed, to our knowledge, only two previous studies have compared CD8^+^ T‐cell responses to different TAA in HCC patients.^12^ Moreover, these studies were limited to analyses of previously described epitopes restricted by human leukocyte antigen (HLA)‐A*02 and HLA‐A*24, respectively.

In this study we used overlapping peptides spanning the entire sequences of AFP, GPC‐3, MAGE‐A1, and NY‐ESO‐1 in a cohort of 96 HCC patients to evaluate naturally occurring CD8^+^ T‐cell responses against four major HCC‐associated TAA irrespective of HLA restriction. Our results provide the first comprehensive view of TAA‐specific CD8^+^ T‐cell responses in this setting with attendant implications for therapeutic vaccine design.

## Materials and Methods

#### Patients and Samples

Patients were recruited from the Department of Internal Medicine and the Department of Surgery at University Hospital Freiburg and from the Department of General and Transplant Surgery at University Hospital Heidelberg. The study was conducted in accordance with the principles of the Declaration of Helsinki under approval and guidance of local Ethics Committees. Ethylenediaminetetraacetate (EDTA)‐anticoagulated blood, pieces of liver biopsies performed for diagnostic purposes, and samples from liver resections were obtained with written informed consent in all cases. Four‐digit HLA‐genotyping was performed using standard techniques.

#### Experimental Procedures

Detailed information on the experimental procedures can be found in the Supporting Online Material.

#### Statistical Analysis

Statistical analyses were performed two‐tailed to a significance level of 95% using GraphPad Prism v. 5 (GraphPad Software, La Jolla, CA). The tests used are indicated in the figure legends. All clinical data were obtained from the date of enrollment. Diagnosis of liver cirrhosis was based on patient charts and sonography, with the addition of histology where available. However, since histology was only available for a fraction of patients, liver cirrhosis cannot be completely excluded in all of the patients classified as noncirrhotic. Progression‐free survival (PFS) was calculated as the number of days between the successful therapeutic intervention closest to enrollment and radiological evidence of disease progression. If patients were lost to follow‐up prior to disease progression, data were censored at the date of the last examination. For the Cox proportional hazards model, IBM SPSS Statistics v. 21 (IBM, Armonk, NY) was used. Lines indicate median values unless stated otherwise.

## Results

#### Study Cohort

A total of 96 HCC patients and 15 controls, including two healthy donors, two patients with acute, and 11 patients with chronic hepatitis were recruited for this study. Cohort characteristics are shown in Table 1 and detailed information on HCC patients is available in Supporting Table 2. Most patients had HCC related to chronic alcohol abuse (35.4%), followed by hepatitis C virus infection (HCV; 24.0%), hepatitis B virus infection (HBV; 9.4%), and nonalcoholic steatohepatitis (NASH; 9.4%). For 17 patients (17.7%) no underlying condition could be identified. Most patients (57.3%) were treatment‐naïve at the time of enrollment, pretreated patients had most commonly received transarterial chemoembolization (TACE; 17.7% of all patients). The patients were staged according to the Barcelona Clinic Liver Cancer (BCLC) classification.^14^ Most patients had early (BCLC A, 28.1%) or intermediate HCC (BCLC B, 36.5%).

**Table 1:** Patient Characteristics

		HCC	Melanoma	Control	Control (T_reg_ Analysis)
Number of patients	96	8	15	61
Gender	% male	84.4	37.5	60	62.3
	% female	14.6	62.5	40	37.7
Age [y]	median	68	46	38	37
	range	45‐85	35‐80	23‐69	22‐73
Serum AFP [ng/ml]	median	11.5	n.d.	n.d.	n.d.
	range	1.4‐60,500			
BCLC stage (%)	0	13.5	n.d.	n.d.	n.d.
	A	28.1			
	B	36.5			
	C	16.7			
	D	4.2			
	unknown	1.0			
Liver cirrhosis (%)	72.9	n.d.	26.7	0
Cause of liver disease (%)	ethanol	35.4	n.d.	6.7	0
	HCV	24.0		40	27.9
	cryptogenic	17.7		13.3	8.2
	HBV	9.4		6.7	14.7
	NASH	9.4		0	1.6
	other	4.2		20	1.6
	healthy	0		13.3	46.0
Treatment prior to inclusion (%)	none	57.3	n.d.	n.d.	n.d.
	TACE	17.7			
	TACE+resection	6.3			
	resection	6.3			
	TACE+RFTA	4.2			
	TACE+sorafenib	1.0			
	PEI	1.0			
	RFTA	1.0			
	unknown	5.2			

Barcelona Clinic Liver Cancer (BCLC) stages: 0, very early; A, early; B, intermediate; C, advanced; D, terminal. HCV: hepatitis C virus. HBV: hepatitis B virus. NASH: nonalcoholic steatohepatitis. TACE: transarterial chemoembolization. RFTA: radiofrequency thermal ablation. PEI: percutaneous ethanol injection. n.d.: not determined.

#### Detection of TAA‐Specific CD8^+^ T‐Cell Responses in Patients With HCC

**Figure 1:**
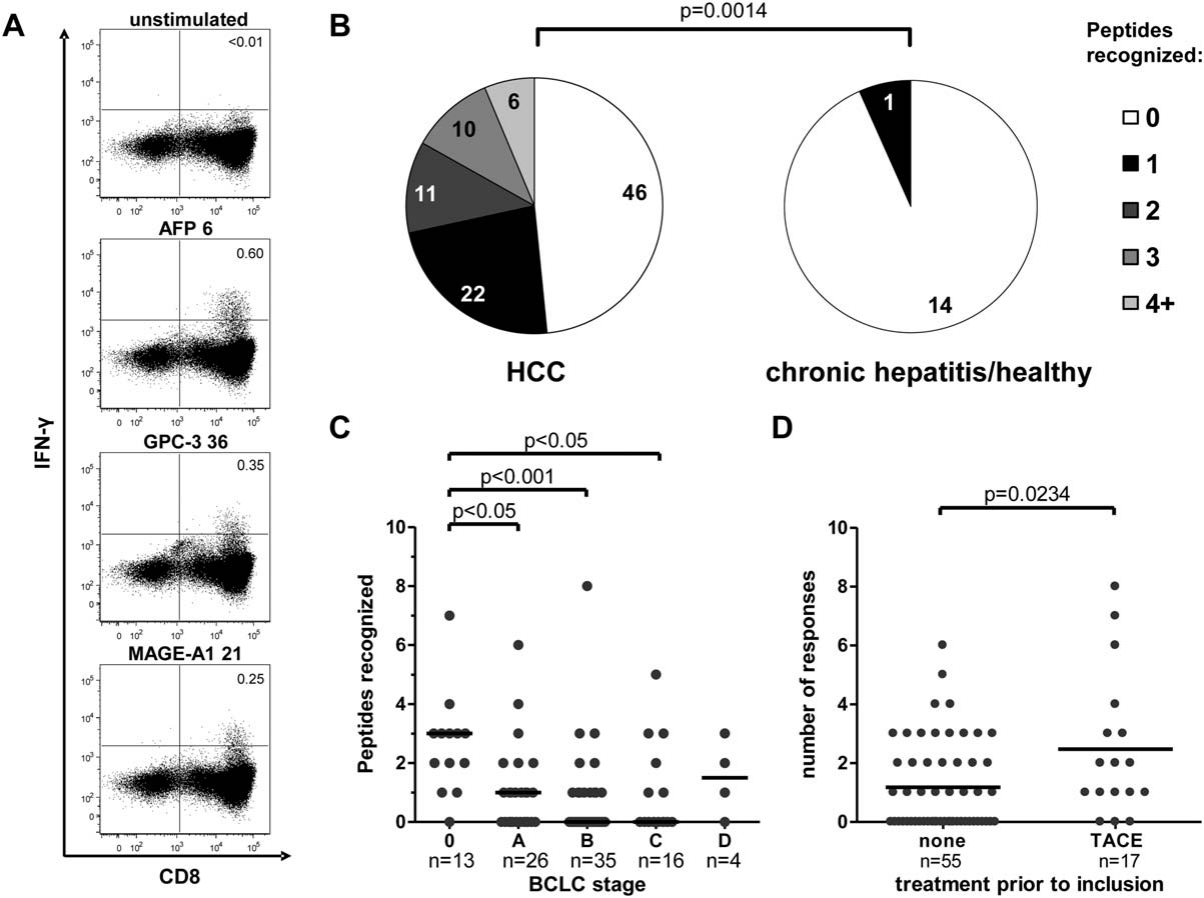
Detection of tumor‐associated antigen‐specific CD8^+^ T cells in patients with HCC. CD8^+^ T cells were isolated from PBMC by magnetic bead enrichment, antigen‐unspecifically expanded and stimulated with overlapping peptides spanning the entire length of AFP, GPC‐3, MAGE‐A1, and NY‐ESO‐1. Production of IFN‐γ was determined by flow cytometry. (A) Representative dotplots showing production of IFN‐γ in response to the indicated peptides in one HCC patient. Plots were gated on live lymphocytes, numbers indicate %IFN‐γ^+^/CD8^+^ T cells. The upper panel shows the unstimulated control. (B) Pie charts comparing the number of CD8^+^ T‐cell responses to the indicated number of peptides in PBMC of 95 HCC patients and 15 controls. The comparison was performed by Mann‐Whitney *U*‐test. (C) The number of individual peptides recognized is shown for HCC patients according to BCLC tumor stage (0, very early; A, early; B, intermediate; C, advanced; D, terminal). Each dot represents one patient. Values were compared by Kruskal‐Wallis test. (D) The number of individual peptides recognized within 55 patients that were treatment‐naïve at the time of inclusion is compared to that of 17 patients that had undergone TACE prior to enrollment. Each dot represents one patient. Values were compared by Mann‐Whitney *U*‐test.

TAA‐specific CD8^+^ T‐cell responses were analyzed by stimulating antigen‐unspecifically expanded CD8^+^ T cells derived from peripheral blood mononuclear cells (PBMC) with overlapping peptides spanning the entire length of AFP, GPC‐3, MAGE‐A1, and NY‐ESO‐1 and then evaluated for the production of interferon‐γ (IFN‐γ) by intracellular cytokine staining and flow cytometry. Representative data are shown in Fig. 1A. TAA‐specific responses were detectable in PBMC of 49 patients (51.6%), most of whom recognized one, two, or three peptides (22, 11, and 10 patients, respectively) while only six patients had responses to four or more different peptides (Fig. 1B). Notably, we were able to detect TAA‐specific CD8^+^ T‐cell responses to all four antigens tested. Only one response, specific for an AFP‐derived peptide, was detected in one individual within the control group. Thus, TAA‐specific CD8^+^ T‐cell responses were significantly more frequently detectable in patients with HCC. Next, we analyzed the association between tumor stage and the presence of TAA‐specific CD8^+^ T cells (Fig. 1C). Significantly more responses were detectable in patients with very early HCC (BCLC 0) compared to patients with later stages (BCLC A, B, or C). There were no differences with regard to response magnitude (%IFN‐γ^+^/CD8^+^ T cells; data not shown). Since locoregional therapy, especially radiofrequency thermal ablation (RFTA) has been described to induce TAA‐specific CD8^+^ T‐cell responses,^15^ we also tested whether we could observe an effect in patients who had undergone locoregional therapy prior to enrollment. We focused on patients pretreated with TACE since they represented the most sizeable population within the pretreated subgroup of the cohort. Indeed, patients previously treated with TACE had significantly more TAA‐specific CD8^+^ T‐cell responses than patients who were treatment‐naïve at the time of enrollment (Fig. 1D).

#### TAA‐Specific CD8^+^ T Cells Infiltrate the Tumor But Do Not Accumulate There

**Figure 2:**
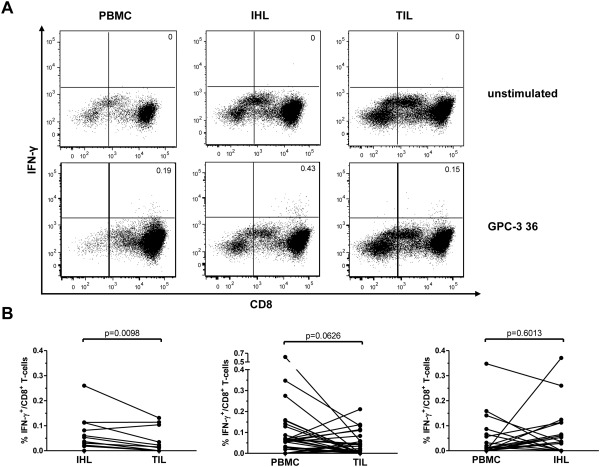
Tumor‐infiltration of TAA‐specific CD8^+^ T cells. CD8^+^ T cells were isolated from PBMC, IHL, and TIL and then evaluated as described in the legend to Fig. 1. (A) Representative dotplots showing the production of IFN‐γ in response to the peptide GPC‐3 36 in one HCC patient in PBMC, IHL, and TIL. Numbers indicate %IFN‐γ^+^/CD8^+^ T cells. The upper panels show the unstimulated control. (B) Pairwise comparison of response magnitude (%IFN‐γ^+^/CD8^+^ T cells) in the different compartments. Each dot represents one peptide‐specific CD8^+^ T‐cell response. Wilcoxon signed rank test was used.

To extend these observations, we analyzed TAA‐specific CD8^+^ T‐cell responses in tumor and surrounding liver tissue. Paired samples of CD8^+^ T cells derived from different sites were available as follows: PBMC and intrahepatic lymphocytes (IHL), 10 patients; PBMC and tumor‐infiltrating lymphocytes (TIL), 17 patients; IHL and TIL, six patients. Samples from all three sites were available for simultaneous analysis from five patients undergoing liver resection. Representative data of one of these patients are shown in Fig. 2A. Even though we were able to detect TAA‐specific CD8^+^ T‐cell responses in all compartments analyzed, a pairwise comparison revealed that TAA‐specific CD8^+^ T‐cell responses were significantly weaker in CD8^+^ T cells derived from TIL compared to those derived from IHL (Fig. 2B). Similarly, TAA‐specific responses in CD8^+^ T cells derived from TIL were weaker than those derived from PBMC, although this trend did not reach statistical significance. Of note, the number of responses did not differ between the individual compartments (data not shown).

#### Presence of TAA‐Specific CD8^+^ T‐Cell Responses Is Associated With Patient Survival

**Figure 3:**
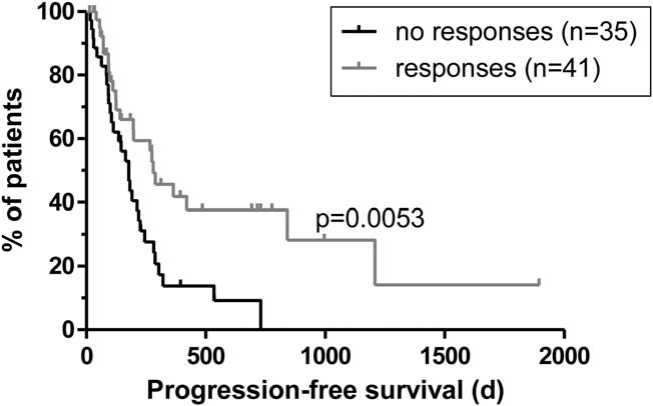
Correlation between TAA‐specific CD8^+^ T‐cell responses and patient survival. PFS data are shown for a total of 76 HCC patients. Median PFS was 177 days in patients without and 279 days in patients with detectable TAA‐specific CD8^+^ T‐cell responses in any of the compartments. Vertical lines indicate censored events. Survival curves were compared by log‐rank test.

For 76 patients, sufficient data from follow‐up examinations at the University Hospital Freiburg was available to enable evaluation of PFS (Supporting Table 2). Of these 76 patients, 41 (53.9%) had detectable TAA‐specific CD8^+^ T‐cell responses. Patients with TAA‐specific responses had a significantly greater median PFS of 279 days compared to 177 days in patients without responses (Fig. 3). Next, we stratified patients according to the number of TAA that triggered CD8^+^ T‐cell responses (Supporting Fig. 1). Indeed, there was a correlation between PFS and the number of TAA recognized. Thus, patient survival appeared to be linked to the occurrence of TAA‐specific CD8^+^ T‐cell responses and their spread across multiple TAA. However, this may be confounded by the observed enrichment of responses in patients with early stage HCC and/or by their association with prior treatments. To address these issues, we established a proportional hazards model for 73 patients with complete datasets (Supporting Table 2). Indeed, this model indicated that BCLC stage, liver cirrhosis, and the recognition of one or two TAA were all independent prognostic factors for PFS (Table 2). Furthermore, resection at the start of PFS (as compared to TACE) and pretreatment with the combination of TACE and RFTA were found as significant contributors. Of note, due to the small number of patients with responses to three or four TAA, the contribution of these subgroups failed to reach statistical significance while still showing a trend for improved survival over patients without responses (Supporting Fig. 1).

**Table 2:** Multivariate Survival Analysis

Parameter	*P*	Hazard ratio	95% confidence interval
BCLC stage			
0	0.015		
A	0.268	2.403	0.590‐11.348
B	0.352	2.127	0.433‐10.439
C	0.011	10.217	1.706‐61.187
Cirrhosis	0.006	3.556	1.433‐8.827
Treatment (PFS)			
TACE	0.320		
RFTA	0.973	0.000	0.000‐1.259 × 10^266^
resection	0.031	0.376	0.155‐0.914
sorafenib	0.770	0.769	0.132‐4.466
RFTA+TACE	0.990	1.016	0.086‐11.939
Prior treatment			
none	0.028		
TACE	0.915	1.050	0.430‐2.565
RFTA	0.204	0.210	0.019‐2.331
resection	0.089	0.302	0.076‐1.201
TACE+resection	0.124	2.283	0.798‐6.535
TACE+RFTA	0.010	0.094	0.016‐0.561
PEI	0.974	27051.681	0.000‐1.372 × 10^275^
TACE+sorafenib	0.170	5.313	0.489‐57.726
TAA recognized			
0	0.024		
1	0.022	0.293	0.102‐0.838
2	0.004	0.172	0.053‐0.565
3	0.294	0.433	0.091‐2.068
4	0.112	0.157	0.016‐1.544

A multivariate Cox proportional hazards model was generated for analysis of progression‐free survival (PFS). This model included data on Barcelona Clinic Liver Cancer (BCLC) stage, treatment received at the start of the PFS interval, treatment received prior to inclusion, liver cirrhosis and the number of tumor‐associated antigens (TAA) triggering CD8^+^ T‐cell responses in a total of 73 patients with complete datasets (Supporting Table 2). These values were either found to correlate with PFS by univariate analysis (BCLC stage, cirrhosis, treatment at start of PFS and number of TAA recognized; data not shown and Supporting Fig. 1) or they correlated with TAA‐specific CD8^+^ T‐cell responses (treatment prior to inclusion, BCLC stage; Fig. 1). Due to the restraints of cohort size, this analysis was limited to those five parameters. *P*‐value, hazard ratio, and its 95% confidence interval for the individual parameters are shown. BCLC stages: 0, very early; A, early; B, intermediate; C, advanced; TACE: transarterial chemoembolization; RFTA: radiofrequency thermal ablation; PEI: percutaneous ethanol injection.

#### Hierarchy of CD8^+^ T‐Cell Responses to Different Tumor Antigens

**Figure 4:**
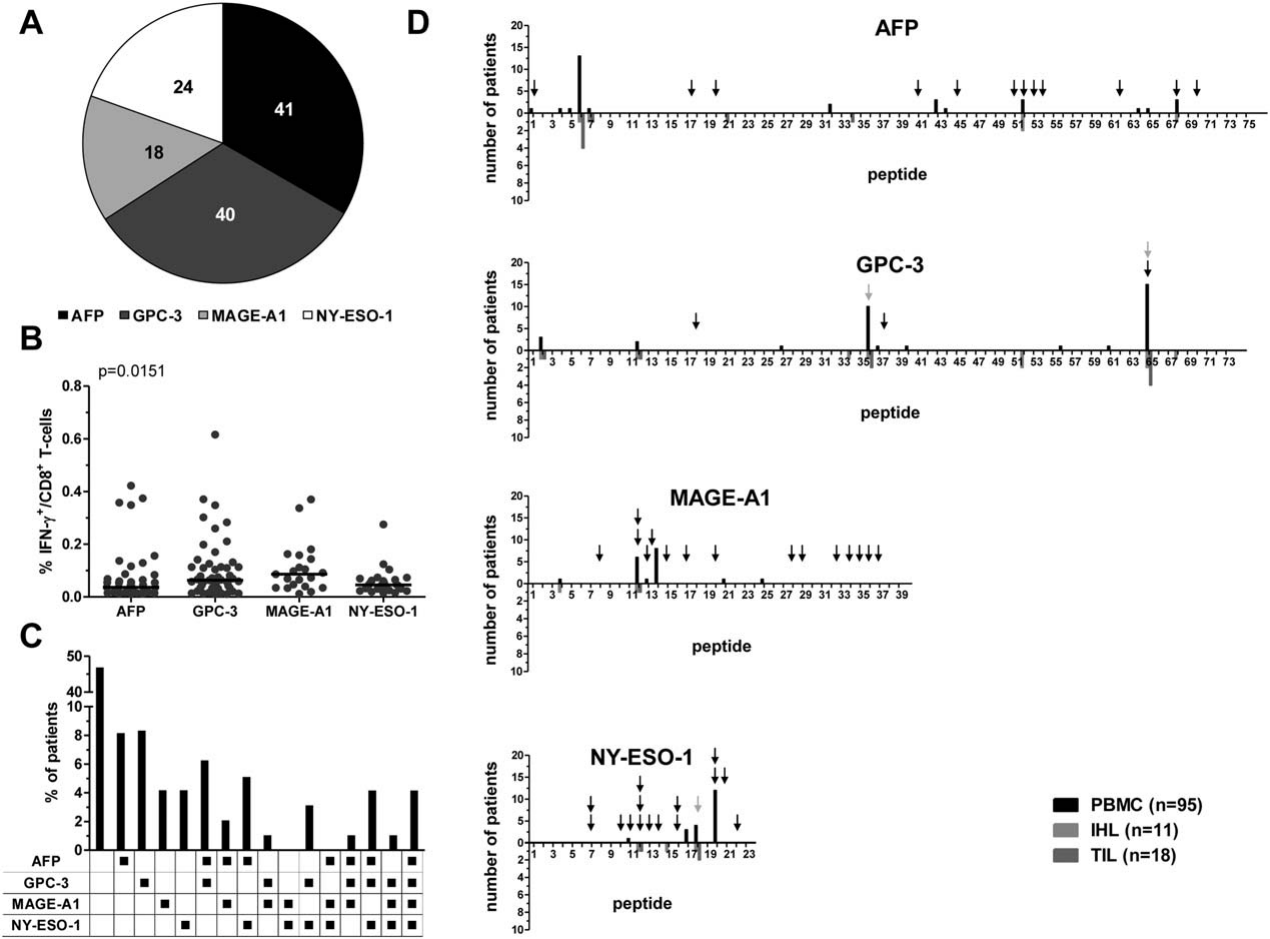
Comparison of CD8^+^ T‐cell responses to different TAA. TAA‐specific CD8^+^ T‐cell responses from PBMC, IHL, and TIL were combined and compared according to the TAA against which they were directed. (A) Pie chart showing the number of responses observed for each TAA. (B) Comparison of TAA‐specific CD8^+^ T‐cell response magnitudes to the different TAA. Each dot represents one peptide‐specific CD8^+^ T‐cell response. The *P*‐value indicated is the overall heterogeneity determined by Kruskal‐Wallis test; posttests did not reveal significant differences between pairs of groups. (C) The individual combinations of TAA recognized in the patient cohort. (D) Immunodominance profiles for each TAA showing the distribution of responses across the individual overlapping peptides. Responses in CD8^+^ T cells derived from PBMC (black) are shown above the x‐axis; responses in CD8^+^ T cells derived from IHL (light gray) and TIL (dark gray) are shown below the x‐axis. Black arrows mark previously described CD8^+^ T‐cell epitopes; gray arrows mark newly fine‐mapped CD8^+^ T‐cell epitopes.

Next, we compared the hierarchy of CD8^+^ T‐cell responses to the individual antigens. Most TAA‐specific CD8^+^ T‐cell responses were observed against AFP and GPC‐3, followed by NY‐ESO‐1 and MAGE‐A1 (Fig. 4A). We also found a significant heterogeneity between the four TAA with respect to the CD8^+^ T‐cell response magnitude (Fig. 4B). The strongest responses were directed against MAGE‐A1 followed by GPC‐3, NY‐ESO‐1, and AFP. As 27 of the 96 patients had responses to more than one antigen, we also asked whether there were any predominant combinations of TAA‐specific CD8^+^ T‐cell responses (Fig. 4C). However, no clear combinatorial associations were apparent.

#### Identification of Immunodominant Regions and Epitopes

To determine immunodominance profiles within the individual TAA, we assessed the distribution of CD8^+^ T‐cell responses across each protein sequence (Fig. 4D). In all four TAA, we identified single dominant regions that were targeted by CD8^+^ T cells. For AFP, this was a previously unmapped region at the N‐terminus of the protein. The immunodominant region of MAGE‐A1 was found in the middle of the protein sequence, and the corresponding regions of GPC‐3 and NY‐ESO‐1 were located at the respective C‐termini. Interestingly, the observed profiles were similar between PBMC, IHL, and TIL, indicating that the same peptides were recognized across different compartments in individual patients (Fig. 4D).

Next, we set out to identify the exact epitopes recognized by TAA‐specific CD8^+^ T cells in patients with HCC. Although several specific epitopes have been described for the TAA analyzed in this study (Fig. 4D, black arrows), only a few of the corresponding overlapping peptides triggered responses in our cohort. Furthermore, some patients with responses to those peptides did not carry the appropriate HLA‐alleles. Responses against overlapping peptides that contained previously identified epitopes are summarized in Supporting Table 3.

To identify novel epitopes, we looked for an enrichment of certain HLA‐alleles, if four or more patients had CD8^+^ T‐cell responses directed against one individual peptide (Supporting Table 3). Subsequently, *in silico* epitope prediction was used to characterize potential epitope candidates. By testing the predicted epitopes in patients carrying the respective HLA‐alleles, we identified three novel CD8^+^ T‐cell epitopes (Fig. 4D, gray arrows). These epitopes are summarized in Supporting Table 3 and exemplary dot plots are shown in Supporting Fig. 2. One epitope was located in the immunodominant region of GPC‐3, while the other two were found in subdominant regions of GPC‐3 and NY‐ESO‐1, respectively.

#### Failure to Generate TAA‐Specific CD8^+^ T‐Cell Lines

In previous work, we failed to generate TAA‐specific CD8^+^ T‐cell lines from HCC patients after *in vitro* stimulation of PBMC with AFP‐derived epitopes restricted by HLA‐A*02. Although peptide‐specific production of IFN‐γ was detectable in antigen‐unspecifically expanded CD8^+^ T cells stimulated with overlapping peptides, no peptide‐specific CD8^+^ T‐cell lines capable of cytokine production could be generated.^7^ In this study, we tried to generate CD8^+^ T‐cell lines specific for the epitopes identified in the screening assay (Supporting Table 3). However, we did not observe epitope‐specific production of IFN‐γ in any of the CD8^+^ T‐cell lines generated, despite the use of epitopes derived from different TAA with various HLA‐restrictions (data not shown).

#### Detection of Dysfunctional TAA‐Specific CD8^+^ T Cells by Tetramer Staining

**Figure 5:**
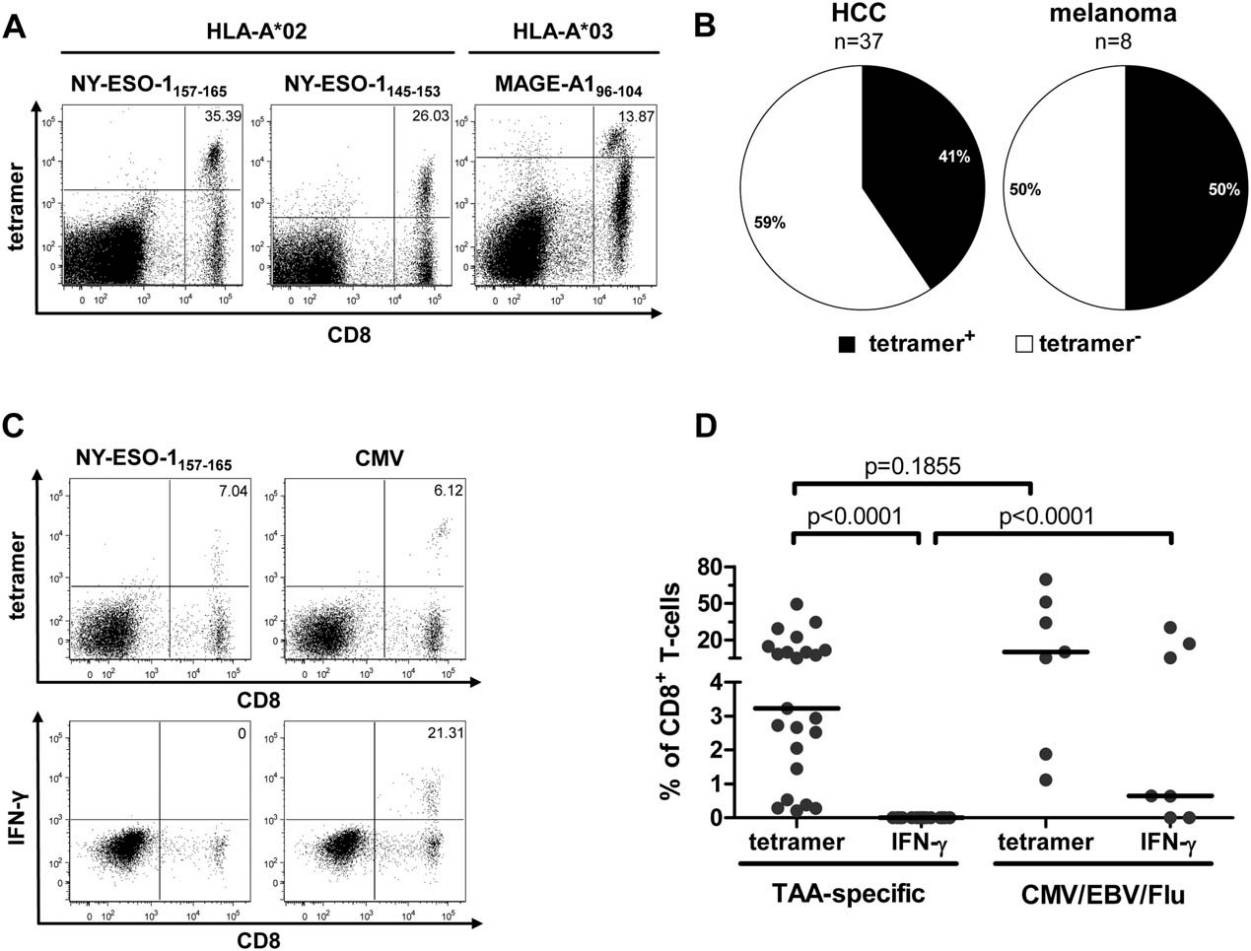
Direct detection of TAA‐specific CD8^+^ T cells by tetramer staining. PBMC isolated from HCC patients were cultured with CD8^+^ T‐cell epitopes restricted by HLA‐A*02 (NY‐ESO‐1_157‐165_ and NY‐ESO‐1_145‐153_) and HLA‐A*03 (MAGE‐A1_96‐104_). After 2 weeks of antigen‐specific expansion, TAA‐specific CD8^+^ T cells were enumerated with tetramers and assessed for IFN‐γ production after peptide stimulation. (A) Representative dotplots showing tetramer stainings of TAA‐specific CD8^+^ T‐cell lines from three different patients. Numbers indicate %tetramer^+^/CD8^+^ T cells. (B) Pie charts showing the frequency of patients with detectable TAA‐specific tetramer^+^CD8^+^ T‐cell populations after expansion. For HCC patients (left), n = 37. For melanoma patients (right), n = 8. (C) Representative tetramer and intracellular IFN‐γ staining data for TAA‐specific and virus‐specific CD8^+^ T‐cell lines from a patient with HCC. Numbers indicate %tetramer^+^/CD8^+^ T cells and %IFN‐γ^+^/CD8^+^ T cells, respectively. (D) Comparison of TAA‐specific and virus‐specific CD8^+^ T‐cell lines. Frequencies of tetramer^+^ and IFN‐γ^+^cells among the CD8^+^ T cells in the culture are shown. Each dot represents a single CD8^+^ T‐cell response. The paired frequencies within TAA‐specific cells were compared by Wilcoxon matched‐pair test, the others by Mann‐Whitney *U*‐test.

Next, we generated tetramers for three epitopes to exclude the possibility that TAA‐specific CD8^+^ T cells proliferated but remained impaired with respect to effector functions and therefore undetectable by intracellular cytokine staining. For this purpose, we chose the well‐characterized immunodominant epitopes NY‐ESO‐1_157‐165_/HLA‐A*02:01 and MAGE‐A1_96‐104_/HLA‐A*03:01 as well as the newly identified epitope NY‐ESO‐1_145‐153_/HLA‐A*02:01. This panel of tetramers allowed us to compare different TAA, HLA‐restrictions, and immunodominance profiles of the corresponding TAA regions. Furthermore, MAGE‐A1 and NY‐ESO‐1 are aberrantly expressed and immunogenic in a variety of malignancies.^17^ Therefore, we included 37 patients with HCC (25 HLA‐A*02^+^, 10 HLA‐A*03^+^, and two HLA‐A*02^+^/A*03^+^) and in addition eight patients with melanoma (five HLA‐A*02^+^, two HLA‐A*03^+^, and one HLA‐A*02^+^/A*03^+^) as controls (Table 1). Indeed, tetramer^+^CD8^+^ T cells were readily detectable after 2 weeks of epitope‐specific expansion in 15 of 37 HCC patients (41%; Fig. 5B), even though we still failed to detect production of IFN‐γ in these cell lines (Fig. 5C,D). Representative data are shown in Fig. 5A. In comparison, four of the eight melanoma patients had detectable TAA‐specific tetramer^+^CD8^+^ T‐cell populations (50%) that also failed to produce IFN‐γ (Fig. 5B; data not shown).

To further characterize these cells, we next analyzed the expression of cytotoxic effector molecules on antigen‐specifically expanded TAA‐specific CD8^+^ T cells from HCC patients. In all cell lines CD25 expression was induced, which is a sign of CD8^+^ T‐cell activation and thus demonstrates antigen encounter (Supporting Fig. 3). However, of the cytotoxic mediators only expression of Fas‐ligand (FasL) was induced consistently, whereas upregulation of granzyme B or perforin was only observed in single cases.

To exclude the possibility of more pervasive immune deficits in HCC patients, we generated control CD8^+^ T‐cell lines, specific for HLA‐A*02‐restricted epitopes derived from cytomegalovirus (CMV), Epstein‐Barr virus (EBV), and influenza virus (flu). In contrast to the corresponding TAA‐specific lines, we found that virus‐specific CD8^+^ T cells derived from HCC patients were capable of producing IFN‐γ after antigen‐specific expansion (Fig. 5C,D). This was not due to a higher frequency of tetramer^+^CD8^+^ T cells in virus‐specific CD8^+^ T‐cell lines, since these frequencies were in a comparable range to those of TAA‐specific CD8^+^ T‐cell lines. We furthermore analyzed the expression of the inhibitory receptors programmed death‐1 (PD‐1) and T‐cell immunoglobulin and mucin‐3 (Tim‐3) after antigen‐specific expansion (Supporting Fig. 4A,B). In this setting, virus‐specific CD8^+^ T‐cell lines strongly expressed PD‐1 but not Tim‐3. In contrast, TAA‐specific CD8^+^ T‐cell lines showed very heterogeneous expression levels of both inhibitory receptors (Supporting Fig. 4C,D). Altogether, TAA‐specific CD8^+^ T cells from HCC patients could be specifically expanded *in vitro* but retained a striking inability to secrete IFN‐γ regardless of the exact nature of the TAA, the immunodominance of the corresponding TAA region, or the restricting HLA‐molecule.

#### T_reg_ Depletion Does Not Restore CD8^+^ T‐Cell Functionality

**Figure 6:**
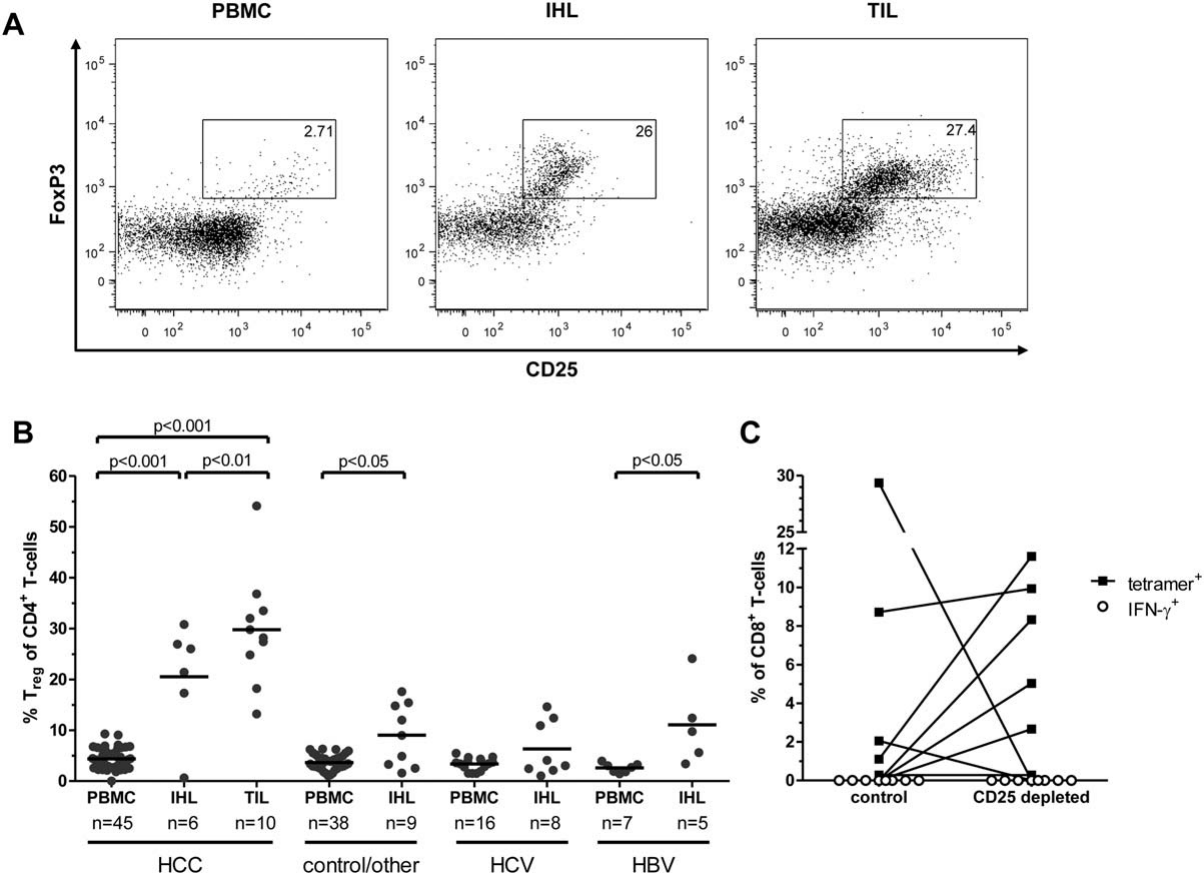
Presence and activity of T_reg_ in patients with HCC. Physical and functional analyses of T_reg_ were conducted using flow cytometry and magnetic bead depletion, respectively. (A) Representative dotplots showing flow cytometric quantification of T_reg_ in PBMC, IHL, and TIL from a patient with HCC. Cells were gated on CD4^+^ events. CD25 and FoxP3 expression are displayed. Numbers indicate percentage of T_reg_ characterized as CD25^+^FoxP3^+^ cells among CD4^+^ T cells. (B) T_reg_ frequencies as in (A) in the indicated compartments of patients with HCC, with chronic viral hepatitis or of controls and patients with other forms of hepatitis. Each dot represents one patient; lines indicate mean values. Frequencies were compared by one‐way analysis of variance (ANOVA). Next to the *P*‐values indicated, frequencies of HCC‐IHL and HCC‐TIL were significantly higher than those of all the other groups (*P* < 0.05 compared to HBV‐IHL and *P* < 0.001 compared to all other groups). (C) Effect of T_reg_‐depletion on TAA‐specific CD8^+^ T‐cell proliferation and function. T_reg_ were removed from PBMC by depletion of CD25^+^ cells using magnetic beads prior to culture. Tetramer‐frequencies (black squares) and production of IFN‐γ (white circles) are indicated.

Finally, we aimed to address the possible mechanisms that might underlie this failure of TAA‐specific CD8^+^ T cells to produce IFN‐γ. Adding a blocking antibody against the ligand of PD‐1 (PD‐L1) at initiation of cultures induced a very weak but detectable production of IFN‐γ in one CD8^+^ T‐cell line and a very strong increase in proliferation in another (Supporting Fig. 5A). However, overall it had no consistent effect. Next, we tested the effects of IL‐7 and IL‐12 at initiation of the cultures, as has been described by others.^13^ This led to an increase in the frequency of TAA‐specific CD8^+^ T cells in 5 of 10 cell lines (50%), while it had an inverse effect in the remaining cell lines. Furthermore, there was no effect on the functionality of TAA‐specific CD8^+^ T cells (Supporting Fig. 5B). As regulatory T cells (T_reg_) are enriched in patients with HCC and can negatively affect CD8^+^ T‐cell function,^18^ we next asked whether these cells were also enriched in our patient cohort. Accordingly, we measured the frequency of T_reg_, defined as CD4^+^CD25^+^FoxP3^+^ cells, in PBMC, IHL, and TIL from HCC patients (Fig. 6A). To enable comparison with noncancer patients, we also studied a large cohort of controls including healthy donors and hepatitis patients undergoing diagnostic liver biopsies (Table 1). T_reg_ were significantly enriched in IHL compared to PBMC (Fig. 6B). This intrahepatic enrichment of T_reg_ was significantly increased in HCC patients, both in IHL and even more so in TIL (Fig. 6B).

As T_reg_ appeared to be a major immunosuppressive mechanism in HCC patients, we depleted CD25^+^ cells (T_reg_) from epitope‐specific CD8^+^ T‐cell lines prior to culture. In five out of eight cell lines (62.5%) with detectable tetramer^+^CD8^+^ T cells we observed an increase in the frequency of tetramer^+^CD8^+^ T cells. In two cases, tetramer^+^CD8^+^ T cells became undetectable after CD25^+^ cell depletion, presumably due to CD25 expression on the TAA‐specific CD8^+^ T cells *ex vivo*. There was no effect on the antigen‐specific production of IFN‐γ, as all cell lines remained dysfunctional (Fig. 6C). Thus, T_reg_ impaired the proliferation of TAA‐specific CD8^+^ T cells in HCC patients. However, T_reg_‐depletion alone did not restore TAA‐specific cytokine production, suggesting that different or additional mechanisms are responsible for the observed dysfunctionality of TAA‐specific CD8^+^ T cells.

## Discussion

In this study we used overlapping peptides spanning the entirety of the AFP, GPC‐3, MAGE‐A1, and NY‐ESO‐1 proteins to conduct a comprehensive and unbiased analysis of immunodominant TAA‐specific CD8^+^ T‐cell responses in patients with HCC. Several important findings emerged from our investigation.

First, antigen‐specific CD8^+^ T‐cell responses directed against all four TAA analyzed were readily observed in more than 50% of HCC patients, significantly greater than the corresponding frequency of detection in healthy donors or patients with viral hepatitis. This is in agreement with our earlier study focused on AFP.^7^ In addition, these TAA‐specific CD8^+^ T‐cell responses were more frequently detectable in patients with very early tumor stage. It is important to note that some of these patients had undergone prior locoregional therapy. In this setting it has been shown that especially RFTA enhances and/or induces TAA‐specific CD8^+^ T‐cell responses in individual patients.^15^ We show that this effect is also detectable for patients treated with TACE. Indeed, it may contribute to the observed enrichment of CD8^+^ T‐cell responses in patients with early stage HCC and supports the view that the release of autologous TAA can induce and/or boost TAA‐specific CD8^+^ T‐cell responses.^15^

Second, the occurrence of TAA‐specific CD8^+^ T‐cell responses in HCC patients was associated with improved PFS. Our results suggest that TAA‐specific CD8^+^ T‐cell responses may indeed be a prognostic factor of patient survival that appears to be independent of the potentially confounding factors of BCLC stage and prior treatment. The mentioned association of strong TAA‐specific CD8^+^ T‐cell responses with PFS in patients undergoing locoregional therapy further supports a possible protective role of such responses.^16^ Especially the occurrence of CD8^+^ T‐cell responses specific for multiple TAA may be beneficial. Even though this aspect requires confirmation in a larger cohort, we found significantly superior PFS in patients with CD8^+^ T‐cell responses to one or two TAA and a similar trend for those with responses to three or four TAA. Furthermore, we observed only one case of tumor progression among the four patients with responses against all four TAA. Based on these data, it is tempting to speculate that TAA‐specific CD8^+^ T‐cell responses may benefit patient survival by hindering tumor growth and thus maintaining early stage disease for longer periods of time.

Third, CD8^+^ T‐cell responses specific for individual TAA displayed clear immunodominance patterns. Immunodominant regions are preferential targets for T‐cell responses because they are immunogenic in most individuals within a cohort.^20^ Indeed, even in our HLA‐independent assay, we observed that most CD8^+^ T‐cell responses targeted single immunodominant regions within a given TAA. Despite our inability to establish a clear immunodominance profile for AFP in a previous study,^7^ we were able to identify an immunodominant region at the N‐terminus by using the larger cohort studied here. In addition, we could identify immunodominant regions in the middle of MAGE‐A1 and at the C‐termini of GPC‐3 and NY‐ESO‐1. It is important to note, however, that the protein regions identified by overlapping peptides in our approach may contain several CD8^+^ T‐cell epitopes restricted by different HLA‐molecules. In this study, almost a third of the overlapping peptides that triggered responses contained previously described CD8^+^ T‐cell epitopes. For example, the immunodominant region of NY‐ESO‐1 contains the epitope NY‐ESO‐1_157‐165_ which is frequently recognized by HLA‐A*02^+^ HCC patients.^12^ However, given that only 58% of patients with a response to the corresponding NY‐ESO‐1‐peptide were HLA‐A*02^+^, it is likely that this region of NY‐ESO‐1 contains additional, unknown CD8^+^ T‐cell epitopes restricted by other HLA‐molecules. Of note, we were able to characterize three novel CD8^+^ T‐cell epitopes within the immunodominant region of GPC‐3 and within subdominant regions of GPC‐3 and NY‐ESO‐1, respectively. Previous studies comparing different TAA focused on epitopes restricted by single HLA‐molecules. For HLA‐A*02, the NY‐ESO‐1_157‐165_ epitope is thought to be immunodominant compared to similarly restricted epitopes derived from other TAA.^12^ However, for HLA‐A*24, no single epitope or TAA was found to be immunodominant.^13^ These results underline the importance of HLA‐independent analyses. Indeed, to our knowledge, this is the first study that compares TAA‐specific CD8^+^ T‐cell responses in HCC patients irrespective of HLA‐restriction. Accordingly, our results have important implications for immunotherapy and tumor vaccine design.

Fourth, functional TAA‐specific CD8^+^ T cells of HCC patients cannot readily be expanded *in vitro*. Indeed, we could not detect peptide‐specific production of IFN‐γ after antigen‐specific culture of CD8^+^ T cells with epitopes derived from MAGE‐A1 and NY‐ESO‐1. These results are in line with previous studies showing that CD8^+^ T‐cell lines specific for either AFP or GPC‐3 fail to produce IFN‐γ.^7^ However, when we used tetramers representing epitopes derived from MAGE‐A1 and NY‐ESO‐1 to detect TAA‐specific CD8^+^ T cells independent of their functionality, we were able to find those cells in more than 50% of HCC patients. The presence of tetramer‐binding TAA‐specific CD8^+^ T cells after *in vitro* expansion, but not *ex vivo* (data not shown), indicates proliferation of a very low frequency precursor population. Thus, cytokine production rather than proliferation may be defective in HCC‐specific CD8^+^ T cells. This is consistent with other studies reporting dysfunctional TAA‐specific CD8^+^ T cells.^8^ It is also a particularly important observation given that the induction of TAA‐specific IFN‐γ production by T cells has been proposed as a major determinant of success in therapeutic tumor vaccine trials targeting other malignancies.^2^ Furthermore, we found a limited expression of cytotoxic mediators by the expanded TAA‐specific CD8^+^ T cells. While FasL was produced, expression of perforin or granzyme B was often not detectable. Indeed, a reduced *ex vivo* expression of perforin and granzyme B among CD8^+^ TIL in HCC patients has been described.^18^ However, in contrast to HCV‐specific CD8^+^ T cells that also lack expression of perforin *ex vivo*, TAA‐specific CD8^+^ T cells were mostly incapable of up‐regulating perforin after antigen‐specific expansion.^21^

Several mechanisms may explain this dysfunctional phenotype of TAA‐specific CD8^+^ T cells. First, the immunosuppressive tumor microenvironment may be responsible, for example, by the production of immunoregulatory cytokines or by the local action of inhibitory receptors such as PD‐1, as described recently in a mouse model.^22^ This is in line with our observation of significantly reduced magnitudes of TAA‐specific CD8^+^ T‐cell responses at the tumor site compared to the periphery. Furthermore, we detected heterogeneous expression of the inhibitory receptors PD‐1 and Tim‐3 on antigen‐specifically expanded TAA‐specific CD8^+^ T cells and effects of PD‐L1 blockade on individual cell lines that were in one case even sufficient to restore the production of IFN‐γ. Second, TAA‐specific T‐cell receptors (TCRs) may have very low affinities for peptide‐HLA complexes relative to virus‐specific TCRs.^24^ Consequently, physiological stimulation of TAA‐specific CD8^+^ T cells with the cognate peptide may deliver a signal that is too weak to restore functionality. This may also explain why IFN‐γ production was preserved after antigen‐independent expansion, which relies on direct crosslinking of CD3ζ rather than TCR engagement. Third, a lack of CD4^+^ T cell help and the action of T_reg_ may also contribute to the observed functional defects.^18^ Indeed, the important role of T_reg_ in the immunobiology of HCC is suggested by the enrichment of these cells in HCC patients, a negative association with patient survival, and the unmasking of AFP‐specific CD4^+^ T‐cell responses after *in vivo* depletion of T_reg_ in HCC patients.^18^ Of note, we also observed T_reg_ enrichment in tumor and liver tissue in HCC patients. In addition, the depletion of T_reg_ prior to antigen‐specific stimulation increased the proliferation of TAA‐specific CD8^+^ T cells in 60% of cases. However, these expanded TAA‐specific CD8^+^ T cells still failed to produce IFN‐γ, suggesting that different or additional mechanisms beyond T_reg_‐mediated effects may contribute to the lack of cytokine production observed in our study and that addressing single pathways contributing to T‐cell failure may be insufficient to restore CD8^+^ T‐cell functionality.

In summary, we have shown that immunodominant TAA‐specific CD8^+^ T‐cell responses in HCC patients are spread across multiple antigens and associated with patient survival. However, the expansion of functional TAA‐specific CD8^+^ T cells from patients with HCC is difficult due to functional impairments. A better understanding of the mechanisms that underlie this dysfunctionality is required to expedite the development of effective immunotherapies for HCC.

## Supplementary Material

Additional Supporting Information may be found in the online version of this article.

Supplementary Figure 1 Correlation between TAA‐specific CD8^+^ T‐cell response breadth and patient survival. PFS data are shown for a total of 76 HCC patients after stratification based on the number of TAA targeted by CD8^+^ T‐cells. The correlation between TAA and PFS was analyzed by logrank‐test for trend.
Supplementary Figure 2 Fine‐mapping of novel CD8^+^ T‐cell epitopes. Antigen‐unspecifically expanded CD8^+^ T‐cells were either left unstimulated, stimulated with overlapping peptides or stimulated with exact epitopes. Production of IFN‐γ is shown in representative dot plots. Each row represents one patient. Peptide sequences are indicated. The exact epitope in the overlapping peptide is underlined. Numbers indicate %IFN‐γ^+^/CD8^+^ T‐cells. (A) Epitope GPC‐3519‐528/HLA‐A*03:01 within peptide GPC‐3 65. (B) Epitope GPC‐3281‐289/HLA‐A*02:01 within peptide GPC‐3 36. (C) Epitope NY‐ESO‐1145‐153/HLA‐A*02:01 within peptide NY‐ESO‐1 18.
Supplementary Figure 3 Expression of cytotoxic mediators after antigen‐specific expansion. After two weeks of antigen‐specific expansion, TAA‐specific CD8^+^ T‐cell lines were analyzed for expression of cytotoxic mediators. Representative dot plots and histogram showing the tetramer‐binding population and its expression of Granzyme B, Perforin, FasL and CD25 (black lines) compared to total CD8^+^ T‐cells (grey) for one patient with a response to MAGE‐A196‐104 (A) and one patient with a response to NY‐ESO‐1157‐165 (B). Numbers indicate %tetramer^+^/CD8^+^ T‐cells. The mean fluorescence intensities (MFI) of tetramer^+^CD8^+^ T‐cells are shown for Granzyme B (C), Perforin (D), FasL (E) and CD25 (F). Each dot represents one of seven TAA‐specific CD8^+^ T‐cell lines. Values were compared by paired t‐test (C‐E) or by Wilcoxon matched‐pair test (F).
Supplementary Figure 4 Expression of inhibitory receptors after antigen‐specific expansion. After two weeks of antigen‐specific expansion, the expression of the inhibitory receptors programmed death‐1 (PD‐1) and T‐cell immunoglobulin and mucin‐3 (Tim‐3) was analyzed on tetramer‐binding CD8^+^ T‐cells. Representative dot plots and histograms showing the tetramer‐binding population and its expression of PD‐1 and Tim‐3 (black lines) compared to total CD8^+^ T‐cells (grey) of one cell line specific for HLA‐A*02/NY‐ESO‐1157‐165 (A) and one specific for HLA‐A*02/EBV BMLF‐1280‐288 (B) are shown. The percentages of PD‐1^+^ (C) and Tim‐3^+^ (D) among tetramer^+^CD8^+^ T‐cells are shown for virus‐specific and TAA‐specific CD8^+^ T‐cell lines. Comparisons were performed by Mann‐Whitney U‐test.
Supplementary Figure 5 Effect of inhibitory receptor blockade and cytokine supplementation on antigen‐specific expansion. Antigen‐specific expansion was essentially performed as described. At initiation of cultures either anti‐PD‐L1 mAb (A) or a mixture of IL‐7 and IL‐12 (B) was added. Black squares represent frequencies of tetramer‐binding cells, white circles represent frequencies of IFN‐γ producing cells. Each dot is representative of one cell line.
