# Supplementary material for: Immunodominance and functional alterations of tumor‐associated antigen‐specific CD8+ T‐cell responses in hepatocellular carcinoma
Source: Hepatology. 2014 Feb 20;59(4):1415–26. doi: 10.1002/hep.26731 (PMC4139003; doi:10.1002/hep.26731)
Supplement: Supplementary file 1 — Supplementary Figure 1 Correlation between TAA‐specific CD8+ T‐cell response breadth and patient survival. PFS data are shown for a total of 76 HCC patients after stratification based on the number of TAA targeted by CD8+ T‐cells. The correlation between TAA and PFS was analyzed by logrank‐test for trend. Supplementary Figure 2 Fine‐mapping of novel CD8+ T‐cell epitopes. Antigen‐unspecifically expanded CD8+ T‐cells were either left unstimulated, stimulated with overlapping peptides or stimulated with exact epitopes. Production of IFN‐γ is shown in representative dot plots. Each row represents one patient. Peptide sequences are indicated. The exact epitope in the overlapping peptide is underlined. Numbers indicate %IFN‐γ+/CD8+ T‐cells. (A) Epitope GPC‐3519‐528/HLA‐A*03:01 within peptide GPC‐3 65. (B) Epitope GPC‐3281‐289/HLA‐A*02:01 within peptide GPC‐3 36. (C) Epitope NY‐ESO‐1145‐153/HLA‐A*02:01 within peptide NY‐ESO‐1 18. Supplementary Figure 3 Expression of cytotoxic mediators after antigen‐specific expansion. After two weeks of antigen‐specific expansion, TAA‐specific CD8+ T‐cell lines were analyzed for expression of cytotoxic mediators. Representative dot plots and histogram showing the tetramer‐binding population and its expression of Granzyme B, Perforin, FasL and CD25 (black lines) compared to total CD8+ T‐cells (grey) for one patient with a response to MAGE‐A196‐104 (A) and one patient with a response to NY‐ESO‐1157‐165 (B). Numbers indicate %tetramer+/CD8+ T‐cells. The mean fluorescence intensities (MFI) of tetramer+CD8+ T‐cells are shown for Granzyme B (C), Perforin (D), FasL (E) and CD25 (F). Each dot represents one of seven TAA‐specific CD8+ T‐cell lines. Values were compared by paired t‐test (C‐E) or by Wilcoxon matched‐pair test (F). Supplementary Figure 4 Expression of inhibitory receptors after antigen‐specific expansion. After two weeks of antigen‐specific expansion, the expression of the inhibitory receptors programmed death‐1 (PD‐1) and T‐cell imm [file hep-59-1415-s7.pdf]

Supplementary Online Material

Materials and Methods

Peptides and tetramers. Comprehensive peptide sets spanning AFP, GPC-3, MAGE-A1 and NY-ESO-1 (18mers overlapping by ten residues; Supplementary Table 1) and individual epitopic peptides were synthesized by Genaxxon (Ulm, Germany), dissolved in DMSO, diluted in RPMI and used at a final concentration of 10 µg/ml. Biotinylated peptide-HLA class I-monomers for the epitopes NY-ESO-$1_{157\text{-}165}$ (SLLMWITQC/HLA-A*02:01), NY-ESO-$1_{145\text{-}153}$ (LQLSISSCL/HLA-A*02:01) and MAGE-$A1_{96\text{-}104}$ (SLFRAVITK/HLA-A*03:01) were generated by refolding the respective heavy chains with β_2-microglobulin in the presence of the respective peptides and subsequently tetramerized with allophycocyanin-conjugated streptavidin (ProZyme, Hayward, CA). Control allophycocyanin-labeled pentamers for the epitopes cytomegalovirus (CMV) $pp65_{495\text{-}503}$ (NLVPMVATV/HLA-A*02:01), Epstein-Barr virus (EBV) BMLF-$1_{280\text{-}288}$ (GLCTLVAML/HLA-A*02:01) and influenza virus $M1_{58\text{-}66}$ (GILGFVFTL/HLA-A*02:01) were obtained from ProImmune (Oxford, UK).

Cell isolation. Peripheral blood mononuclear cells (PBMC) were isolated by density gradient centrifugation with Pancoll separation medium (PAN Laboratories, Eidenbach, Germany) and washed two times with phosphate-buffered saline (PBS). Intrahepatic lymphocytes (IHL) and tumor-infiltrating lymphocytes (TIL) were obtained by gently grinding liver and tumor material through a sterile 70 nm cell strainer (BD Biosciences, Franklin Lakes, NJ). Cells isolated from biopsies were used directly, cells from surgical resections were subjected to density gradient centrifugation prior to use.

CD8⁺ T-cell selection and culture. CD8$^+$ T-cells were isolated by incubating 4x10^6 PBMC with anti-CD8 monoclonal antibody (mAb)-coated magnetic beads and subsequently separating the cells using a magnetic holder (both Life Technologies, Darmstadt, Germany). Purity of CD8$^+$ T-cells was >95% as determined by flow cytometry. CD8$^+$ T-cells were plated into one well of a 24-well plate (Greiner Bio One, Frickenhausen, Germany) in 2 ml complete medium (RPMI with 10% fetal bovine serum, 1% penicillin/streptomycin and 1.5% 1 M HEPES; all Life technologies) together with 2x10^6 irradiated autologous PBMC and supplemented with 100 U/ml recombinant human IL-2 (Hoffmann-La Roche, Basel, Switzerland) and 0.04 µg/ml anti-CD3 mAb (Immunotech, Marseilles, France). Twice a week for three weeks, cells were split and supplemented with fresh medium containing IL-2 at a final concentration of 100 U/ml. Experiments were performed no earlier than four days after the last addition of IL-2.

Generation of epitope-specific CD8$^+$ T-cell lines. CD8$^+$ T-cell lines were generated by plating 4x10^6 PBMC in 1 ml complete medium into one well of a 24-well plate in the presence of 10 µg/ml of the respective peptide and 0.5 µg/ml anti-CD28 mAb (BD). 1 ml complete medium containing recombinant human IL-2 at a final concentration of 20 U/ml was added on days 4 and 11. On day 7, the supernatant was removed and the cells were resuspended in 1 ml complete medium containing 2x10^6 irradiated autologous PBMC and 10 µg/ml of the respective peptide. In some experiments 10 µg/ml anti-PD-L1 mAb (eBioscience, San Diego, CA) or a combination of 10 ng/ml IL-7 (R&D Systems, Minneapolis, MN) and 100 pg/ml IL-12 (PeproTech, Rocky Hill, CT) was added at initiation of cultures. Experiments were performed on day 14 of culture.

Depletion of regulatory T-cells (T_{reg}). PBMC were labeled with anti-CD25 magnetic beads and $CD25^+$ cells depleted using a magnetic holder, as described above for $CD8^+$ T-cell isolation. Epitope-specific $CD8^+$ T-cell lines were then generated as described above.

Tetramer-staining. $1x10^6$ cells were incubated in one well of a 96-well V-bottom plate (Greiner Bio One) with the respective tetramer in PBS containing 1% fetal bovine serum at 37 °C for 15 min. Subsequently, cells were washed, blocked with pure mouse immunoglobulin G1 (BD), and then surface-stained with phycoerythrin (PE)-labeled anti-CD8 mAb and 7-amino-actinomycin D (ViaProbe; both BD). Finally, cells were fixated in PBS/2% paraformaldehyde. In some experiments, PE-Cy7-labeled anti-PD-1 and PE-labeled anti-Tim-3 mAbs (both BioLegend, San Diego, CA) were used additionally for surface-staining. In this case, allophycocyanin-H7-labeled anti-CD8 mAb (BD) was used.

Intracellular cytokine staining. For intracellular cytokine staining, cells were incubated for 5 h at 37 °C in one well of a 96-well V-bottom plate in the presence of 50 U/ml recombinant human IL-2 and 1 µg/ml Brefeldin A (BD). Unstimulated wells received no further additions. For peptide-stimulation, 10 µg/ml of the respective peptide (pools) were added. A combination of 10 ng/ml phorbol-12-myristate-13-acetate and 0.2 µg/ml ionomycin (both Sigma-Aldrich, Seelze, Germany) was used as positive control. Following incubation, cells were surface-stained with PE-labeled anti-CD8 mAb and ViaProbe and permeabilized using Cytofix/Cytoperm (all BD). Subsequently, the cells were washed with Perm/Wash buffer (BD), stained intracellularly with fluorescein-isothiocyanate-labeled anti-interferon-γ mAb (BD) and fixated with PBS/2% paraformaldehyde. Samples were stored over night at 4 °C prior to flow cytometric acquisition.

Regulatory T-cell (T_{reg}) staining. PBMC, IHL or TIL were surface stained with peridinin-chlorophyll-protein-labeled anti-CD4 (BD) and allophycocyanin-labeled anti-CD25 (eBioscience) mAbs. Cells were then permeabilized with the FoxP3/Transcription-Factor-Staining-Buffer-Set (eBioscience) and stained with AlexaFluor488-labeled anti-FoxP3 mAb (eBioscience) according to the manufacturer's instructions. Stained samples were analyzed by flow cytometry on the same day after fixation with PBS/2% paraformaldehyde.

Perforin staining. Antigen-specifically expanded cells were stained with tetramer as described above and surface stained with allophycocyanin-H7-labeled anti-CD25, V500-labeled anti-CD8 mAbs and ViaProbe (all BD). Cells were then permeabilized as described for T_{reg} staining and stained with fluorescein-isothiocyanate-labeled anti-Granzyme B, biotin-labeled anti-FasL (both BD) and PE-labeled anti-Perforin (Diaclone, Besançon, France) mAbs according to the manufacturers' instructions. Subsequently, cells were stained with Streptavidin-eFluor450 (eBioscience). Stained samples were analyzed by flow cytometry on the same day after fixation with PBS/2% paraformaldehyde.

Flow cytometry. Samples were acquired using a BD FACSCanto II flow cytometer (BD). Data were analyzed using FlowJo software (Treestar, Inc., Ashland, OR). Dead cells (ViaProbe$^+$) were excluded from analyses.

Data analysis. Flow cytometry data were analyzed using FlowJo software (Treestar, Inc., Ashland, OR). Screening was performed by stimulating antigen-unspecifically expanded CD8$^+$ T-cells with pools of five peptides each. The pools that triggered production of interferon-γ (IFN-γ) were broken down to individual peptides which were then evaluated in triplicate after subtraction of unstimulated background values. Response frequencies were calculated as the mean of the %IFN-γ$^+$/CD8$^+$ cells of each triplicate. Responses with a minimum of 0.01% were considered positive. For tetramer stainings, gates were set individually for each patient according to tetramer-unstained control wells.

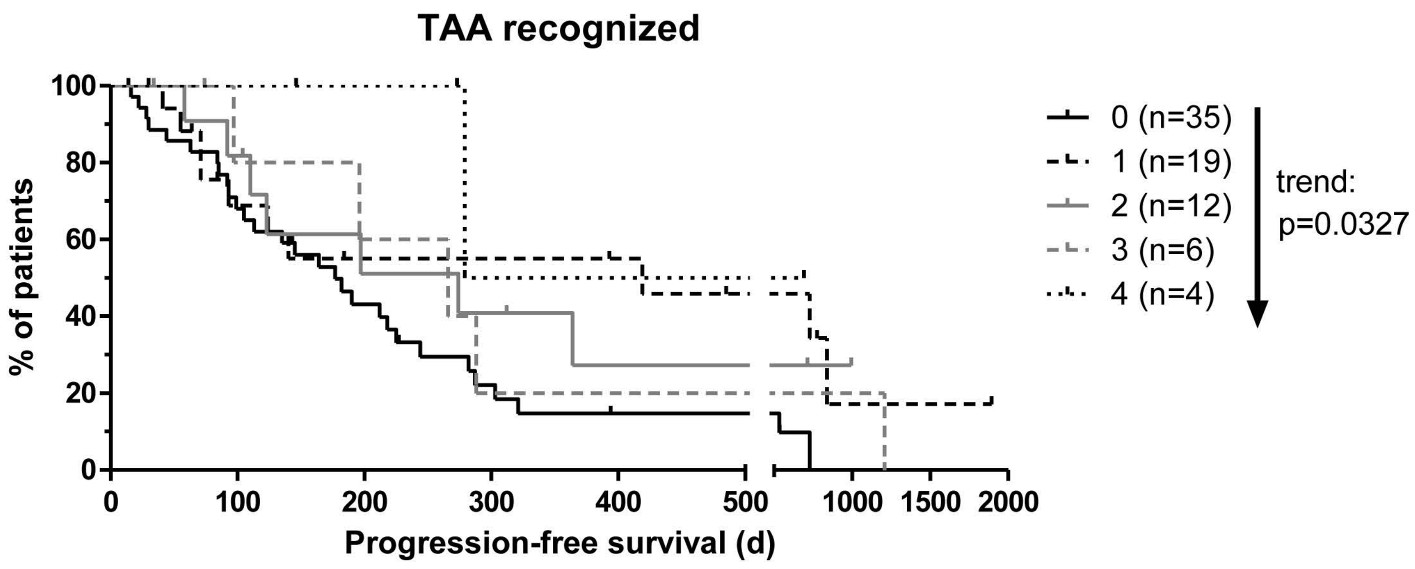

TAA recognized
100
80
60
40
20
0
% of patients
0
100
200
300
400
500
1000
1500
2000
Progression-free survival (d)
0 (n=35)
1 (n=19)
2 (n=12)
3 (n=6)
4 (n=4)
trend:
p=0.0327

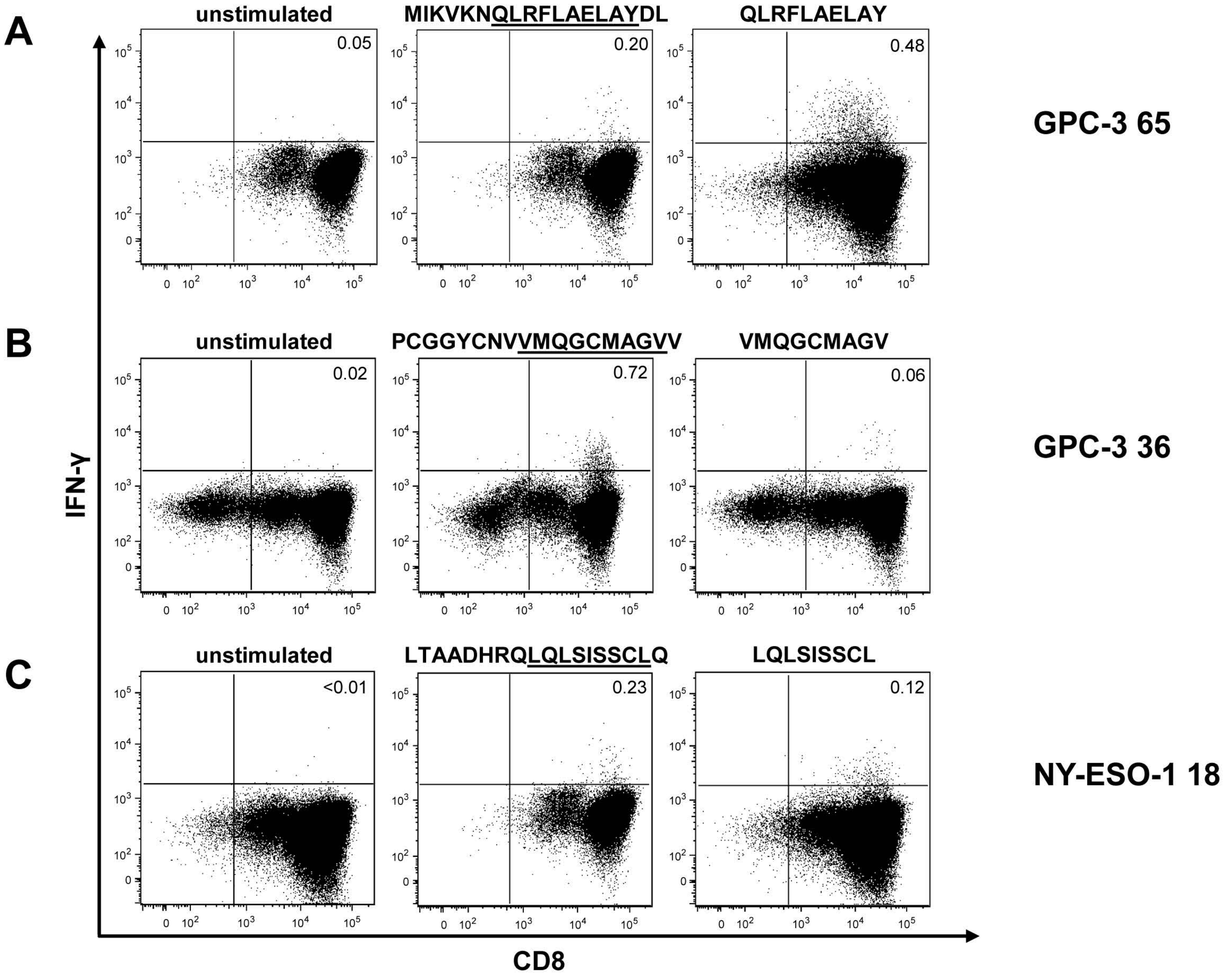
A
unstimulated
0.05
MIKVKNQLRFLAELAYDL
0.20
QLRFLAELAY
0.48
GPC-3 65
B
unstimulated
0.02
PCGGYCNVVMQGCMAGVV
0.72
VMQGCMAGV
0.06
GPC-3 36
C
unstimulated
<0.01
LTAADHRQLQLSISSCLQ
0.23
LQLSISSCL
0.12
NY-ESO-1 18
IFN-γ
CD8

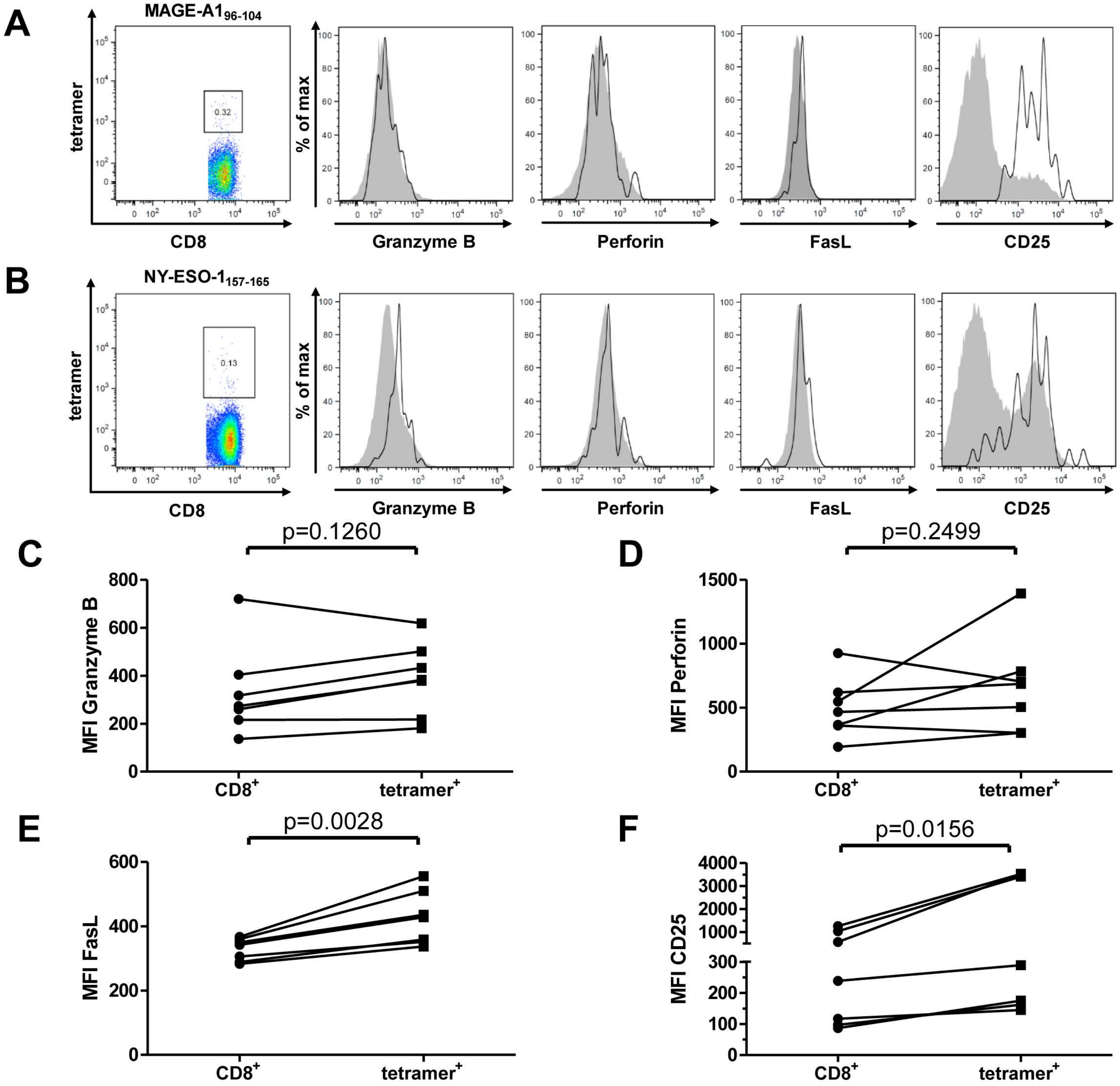

A
MAGE-A1$_{96-104}$
tetramer
CD8
0.32
% of max
Granzyme B
Perforin
FasL
CD25
B
NY-ESO-1$_{157-165}$
0.13
C
p=0.1260
MFI Granzyme B
CD8$^+$
tetramer$^+$
D
p=0.2499
MFI Perforin
E
p=0.0028
MFI FasL
F
p=0.0156
MFI CD25

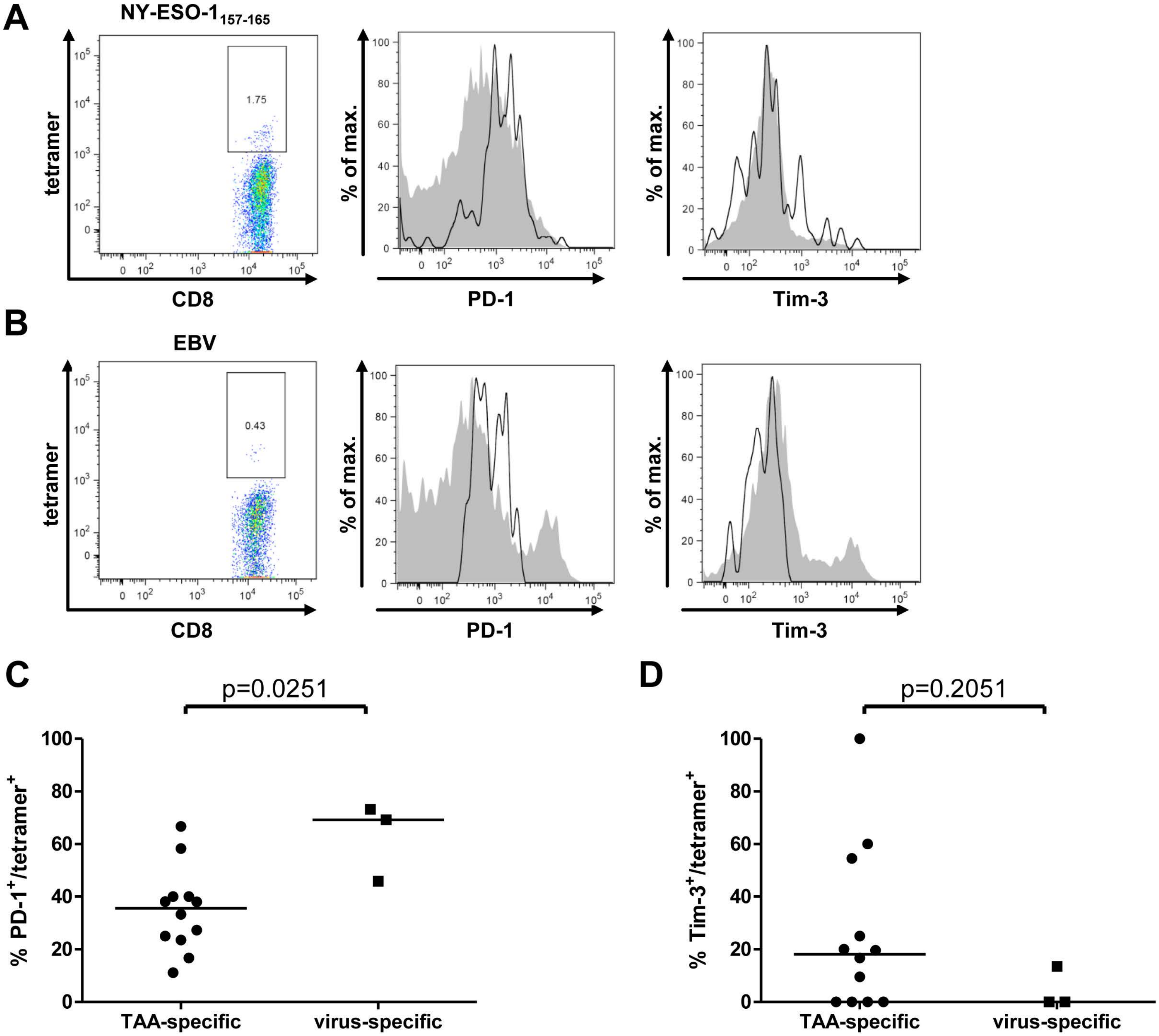
A
NY-ESO-1157-165
1.75
tetramer
CD8
% of max.
PD-1
% of max.
Tim-3
B
EBV
0.43
tetramer
CD8
% of max.
PD-1
% of max.
Tim-3
C
p=0.0251
% PD-1+/tetramer+
TAA-specific
virus-specific
D
p=0.2051
% Tim-3+/tetramer+
TAA-specific
virus-specific

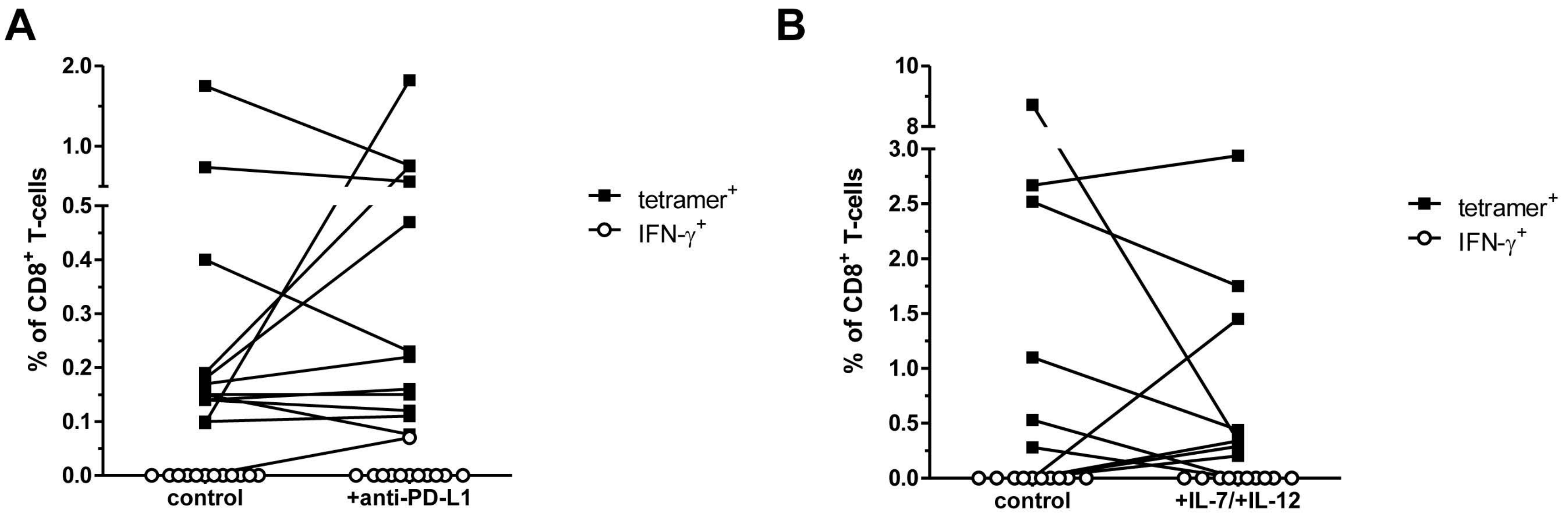

A
% of CD8+ T-cells
2.0
1.0
0.5
0.4
0.3
0.2
0.1
0.0
control
+anti-PD-L1
tetramer+
IFN-γ+
B
% of CD8+ T-cells
10
8
3.0
2.5
2.0
1.5
1.0
0.5
0.0
control
+IL-7/+IL-12
tetramer+
IFN-γ+

Supplementary Table 1 List of overlapping peptides.

The sequences of the overlapping peptides for each of the four TAAs and their position within the protein sequence are shown. aa: amino acid

	**AFP**		
**peptide**	**sequence**	**start (aa)**	**end (aa)**
1	MKWVESIFLIFLLNFTES	1	18
2	LIFLLNFTESRTLHRNEY	9	26
3	ESRTLHRNEYGIASILDS	17	34
4	EYGIASILDSYQCTAEIS	25	42
5	DSYQCTAEISLADLATIF	33	50
6	ISLADLATIFFAQFVQEA	41	58
7	IFFAQFVQEATYKEVSKM	49	66
8	EATYKEVSKMVKDALTAI	57	74
9	KMVKDALTAIEKPTGDEQ	65	82
10	AIEKPTGDEQSSGCLENQ	73	90
11	EQSSGCLENQLPAFLEEL	81	98
12	NQLPAFLEELCHEKEILE	89	106
13	ELCHEKEILEKYGHSDCC	97	114
14	LEKYGHSDCCSQSEEGRH	105	122
15	CCSQSEEGRHNCFLAHKK	113	130
16	RHNCFLAHKKPTPASIPL	121	138
17	KKPTPASIPLFQVPEPVT	129	146
18	PLFQVPEPVTSCEAYEED	137	154
19	VTSCEAYEEDRETFMNKF	145	162
20	EDRETFMNKFIYEIARRH	153	170
21	KFIYEIARRHPFLYAPTI	161	178
22	RHPFLYAPTILLWAARYD	169	186
23	TILLWAARYDKIIPSCCK	177	194
24	YDKIIPSCCKAENAVECF	185	202
25	CKAENAVECFQTKAATVT	193	210
26	CFQTKAATVTKELRESSL	201	218
27	VTKELRESSLLNQHACAV	209	226
28	SLLNQHACAVMKNFGTRT	217	234
29	AVMKNFGTRTFQAITVTK	225	242
30	RTFQAITVTKLSQKFTKV	233	250
31	TKLSQKFTKVNFTEIQKL	241	258
32	KVNFTEIQKLVLDVAHVH	249	266
33	KLVLDVAHVHEHCCRGDV	257	274
34	VHEHCCRGDVLDCLQDGE	265	282
35	DVLDCLQDGEKIMSYICS	273	290
36	GEKIMSYICSQQDTLSNK	281	298
37	CSQQDTLSNKITECCKLT	289	306
38	NKITECCKLTTLERGQCI	297	314
39	LTTLERGQCIIHAENDEK	305	322
40	CIIHAENDEKPEGLSPNL	313	330
41	EKPEGLSPNLNRFLGDRD	321	338
42	NLNRFLGDRDFNQFSSGE	329	346
43	RDFNQFSSGEKNIFLASF	337	354
44	GEKNIFLASFVHEYSRRH	345	362

45	SFVHEYSRRHPQLAVSVI	353	370
46	RHPQLAVSVILRVAKGYQ	361	378
47	VILRVAKGYQELLEKCFQ	369	386
48	YQELLEKCFQTENPLECQ	377	394
49	FQTENPLECQDKGEEELQ	385	402
50	CQDKGEEELQKYIQESQA	393	410
51	LQKYIQESQALAKRSCGL	401	418
52	QALAKRSCGLFQKLGEYY	409	426
53	GLFQKLGEYYLQNAFLVA	417	434
54	YYLQNAFLVAYTKKAPQL	425	442
55	VAYTKKAPQLTSSELMAI	433	450
56	QLTSSELMAITRKMAATA	441	458
57	AITRKMAATAATCCQLSE	449	466
58	TAATCCQLSEDKLLACGE	457	474
59	SEDKLLACGEGAADIIIG	465	482
60	GEGAADIIIGHLCIRHEM	473	490
61	IGHLCIRHEMTPVNPGVG	481	498
62	EMTPVNPGVGQCCTSSYA	489	506
63	VGQCCTSSYANRRPCFSS	497	514
64	YANRRPCFSSLVVDETYV	505	522
65	SSLVVDETYVPPAFSDDK	513	530
66	YVPPAFSDDKFIFHKDLC	521	538
67	DKFIFHKDLCQAQGVALQ	529	546
68	LCQAQGVALQTMKQEFLI	537	554
69	LQTMKQEFLINLVKQKPQ	545	562
70	LINLVKQKPQITEEQLEA	553	570
71	PQITEEQLEAVIADFSGL	561	578
72	EAVIADFSGLLEKCCQGQ	569	586
73	GLLEKCCQGQEQEVCFAE	577	594
74	GQEQEVCFAEEGQKLSIK	585	602
75	FAEEGQKLISKTRAALGV	593	610

	GPC-3		
**peptide**	**sequence**	**start (aa)**	**end (aa)**
1	MAGTVRTACLVVAMLLSL	1	18
2	CLVVAMLLSLDFPGQAQP	9	26
3	SLDFPGQAQPPPPPPDAT	17	34
4	QPPPPPPDATCHQVRSFF	25	42
5	ATCHQVRSFFQRLQPGLK	33	50
6	FFQRLQPGLKWVPETPVP	41	58
7	LKWVPETPVPGSDLQVCL	49	66
8	VPGSDLQVCLPKGPTCCS	57	74
9	CLPKGPTCCSRKMEEKYQ	65	82
10	CSRKMEEKYQLTARLNME	73	90
11	YQLTARLNMEQLLQSASM	81	98
12	MEQLLQSASMELKFLIIQ	89	106
13	SMELKFLIIQNAAVFQEA	97	114
14	IQNAAVFQEAFEIVVRHA	105	122
15	EAFEIVVRHAKNYTNAMF	113	130
16	HAKNYTNAMFKNNYPSLT	121	138
17	MFKNNYPSLTPQAFEFVG	129	146
18	LTPQAFEFVGEFFTDVSL	137	154
19	VGEFFTDVSLYILGSDIN	145	162
20	SLYILGSDINVDDMVNEL	153	170
21	INVDDMVNELFDSLFPVI	161	178
22	ELFDSLFPVIYTQLMNPG	169	186
23	VIYTQLMNPGLPDSALDI	177	194
24	PGLPDSALDINECLRGAR	185	202
25	DINECLRGARRDLKVFGN	193	210
26	ARRDLKVFGNFPKLIMTQ	201	218
27	GNFPKLIMTQVSKSLQVT	209	226
28	TQVSKSLQVTRIFLQALN	217	234
29	VTRIFLQALNLGIEVINT	225	242
30	LNLGIEVINTTDHLKFSK	233	250
31	NTTDHLKFSKDCGRMLTR	241	258
32	SKDCGRMLTRMWYCSYCQ	249	266
33	TRMWYCSYCQGLMMVKPC	257	274
34	CQGLMMVKPCGGYCNVVM	265	282
35	PCGGYCNVVMQGCMAGVV	273	290
36	VMQGCMAGVVEIDKYWRE	281	298
37	VVEIDKYWREYILSLEEL	289	306
38	REYILSLEELVNGMYRIY	297	314
39	ELVNGMYRIYDMENVLLG	305	322
40	IYDMENVLLGLFSTIHDS	313	330
41	LGLFSTIHDSIQYVQKNA	321	338
42	DSIQYVQKNAGKLTTTIG	329	346
43	NAGKLTTTIGKLCAHSQQ	337	354
44	IGKLCAHSQQRQYRSAYY	345	362
45	QQRQYRSAYYPEDLFIDK	353	370
46	YYPEDLFIDKKVLKVAHV	361	378
47	DKKVLKVAHVEHEETLSS	369	386
48	HVEHEETLSSRRRELIQK	377	394
49	SSRRRELIQKLKSFISFY	385	402

50	QKLKSFISFYSALPGYIC	393	410
51	FYSALPGYICSHSPVAEN	401	418
52	ICSHSPVAENDTLCWNGQ	409	426
53	ENDTLCWNGQELVERYSQ	417	434
54	GQELVERYSQKAARNGMK	425	442
55	SQKAARNGMKNQFNLHEL	433	450
56	MKNQFNLHELKMKGPEPV	441	458
57	ELKMKGPEPVVSQIIDKL	449	466
58	PVVSQIIDKLKHINQLLR	457	474
59	KLKHINQLLRTMSMPKGR	465	482
60	LRTMSMPKGRVLDKNLDE	473	490
61	GRVLDKNLDEEGFESGDC	481	498
62	DEEGFESGDCGDDEDECI	489	506
63	DCGDDEDECIGGSGDGMI	497	514
64	CIGGSGDGMIKVKNQLRF	505	522
65	MIKVKNQLRFLAELAYDL	513	530
66	RFLAELAYDLDVDDAPGN	521	538
67	DLDVDDAPGNSQQATPKD	529	546
68	GNSQQATPKDNEISTFHN	537	554
69	KDNEISTFHNLGNVHSPL	545	562
70	HNLGNVHSPLKLLTSMAI	553	570
71	PLKLLTSMAISVVCFFFL	561	578
72	AISVVCFFFLVHMAGTVR	569	6
73	FLVHMAGTVRTACLVVAM	577	14

MAGE-A1

peptide	sequence	start (aa)	end (aa)
1	MSLEQRSLHCKPEEALEA	1	18
2	HCKPEEALEAQQEALGLV	9	26
3	EAQQEALGLVCVQAATSS	17	34
4	LVCVQAATSSSSPLVLGT	25	42
5	SSSSPLVLGTLEEVPTAG	33	50
6	GTLEEVPTAGSTDPPQSP	41	58
7	AGSTDPPQSPQGASAFPT	49	66
8	SPQGASAFPTTINFTRQR	57	74
9	PTTINFTRQRQPSEGSSS	65	82
10	QRQPSEGSSSREEEGPST	73	90
11	SSREEEGPSTSCILESLF	81	98
12	STSCILESLFRAVITKKV	89	106
13	LFRAVITKKVADLVGFLL	97	114
14	KVADLVGFLLLKYRAREP	105	122
15	LLLKYRAREPVTKAEMLE	113	130
16	EPVTKAEMLESVIKNYKH	121	138
17	LESVIKNYKHCFPEIFGK	129	146
18	KHCFPEIFGKASESLQLV	137	154
19	GKASESLQLVFGIDVKEA	145	162
20	LVFGIDVKEADPTGHSYV	153	170
21	EADPTGHSYVLVTCLGLS	161	178
22	YVLVTCLGLSYDGLLGDN	169	186
23	LSYDGLLGDNQIMPKTGF	177	194
24	DNQIMPKTGFLIIVLVMA	185	202
25	GFLIIVLVMAMEGGHAPE	193	210
26	MAMEGGHAPEEEIWEELS	201	218
27	PEEEIWEELSVMEVYDGR	209	226
28	LSVMEVYDGREHSAYGEP	217	234
29	GREHSAYGEPRKLLTQDL	225	242
30	EPRKLLTQDLVQEKYLEY	233	250
31	DLVQEKYLEYRQVPDSDP	241	258
32	EYRQVPDSDPARYEFLWG	249	266
33	DPARYEFLWGPRALAETS	257	274
34	WGPRALAETSYVKVLEYV	265	282
35	TSYVKVLEYVIKVSARVR	273	290
36	YVIKVSARVRFFFPSLRE	281	298
37	VRFFFPSLREAALREEEE	289	306
38	REAALREEEEGVMSLEQR	297	6
39	EEGVMSLEQRSLHCKPEE	305	14

	**NY-ESO-1**		
**peptide**	**sequence**	**start (aa)**	**end (aa)**
1	MQAEGRGTGGSTGDADGP	1	18
2	GGSTGDADGPGGPGIPDG	9	26
3	GPGGPGIPDGPGGNAGGP	17	34
4	DGPGGNAGGPGEAGATGG	25	42
5	GPGEAGATGGRGPRGAGA	33	50
6	GGRGPRGAGAARASGPGG	41	58
7	GAARASGPGGGAPRGPHG	49	66
8	GGGAPRGPHGGAASGLNG	57	74
9	HGGAASGLNGCCRCGARG	65	82
10	NGCCRCGARGPESRLLEF	73	90
11	RGPESRLLEFYLAMPFAT	81	98
12	EFYLAMPFATPMEAELAR	89	106
13	ATPMEAELARRSLAQDAP	97	114
14	ARRSLAQDAPPLPVPGVL	105	122
15	APPLPVPGVLLKEFTVSG	113	130
16	VLLKEFTVSGNILTIRLT	121	138
17	SGNILTIRLTAADHRQLQ	129	146
18	LTAADHRQLQLSISSCLQ	137	154
19	LQLSISSCLQQLSLLMWI	145	162
20	LQQLSLLMWITQCFLPVF	153	170
21	WITQCFLPVFLAQPPSGQ	161	178
22	VFLAQPPSGQRRMQAEGR	169	6
23	GQRRMQAEGRGTGGSTGD	177	14

Supplementary Table 2 Detailed patient characteristics

Detailed information on the patients included in the study. For patient 18 no peripheral blood mononuclear cells (PBMC) were available, thus yielding a total of 95 patients for analyses based on PBMC. Patients that were not eligible for analysis of progression-free survival (PFS) because of lacking follow-up examinations are indicated by the sign not available (n/a) in the PFS section. The treatment at the start of each PFS interval is indicated as well as the treatment the patients had received prior to inclusion into the study. For multivariate survival analyses, patients with incomplete datasets were excluded (marked in grey). IHL: intrahepatic lymphocytes. TIL: tumor-infiltrating lymphocytes. M: male. F: female. y: years. LC: liver cirrhosis. HCV: hepatitis C virus. NASH: non-alcoholic steatohepatitis. HBV: hepatitis B virus. AFP: α-fetoprotein. BCLC: Barcelona clinic liver cancer stage. RFTA: radio-frequency thermal ablation. TACE: trans-arterial chemo-embolization. PEI: percutaneous ethanol-injection. d: days. cens.: censored. resp.: number of responses. TAA: number of tumor-associated antigens recognized.

#	samples	sex	age [y]	LC	etiology	AFP [ng/ml]	BCLC	prior treatment	treatment PFS	PFS [d]	cens.	resp.	TAA
**1**	PBMC	M	75	yes	ethanol	4.6	A	TACE	TACE	196	no	4	3
**2**	PBMC	F	60	yes	ethanol	2.3	C	none	n/a	n/a	n/a	3	2
**3**	PBMC	M	77	yes	HCV	20.0	A	RFTA+ TACE	TACE	244	no	0	0
**4**	PBMC	M	52	no	NASH	97.9	C	resection+ TACE	TACE	99	no	0	0
**5**	PBMC	M	68	yes	ethanol	60,500	C	none	TACE	93	no	1	1
**6**	PBMC	M	71	yes	ethanol	3.8	0	TACE	TACE	364	no	3	2
**7**	PBMC+TIL	M	84	yes	ethanol	6.0	D	none	n/a	n/a	n/a	3	2
**8**	PBMC	M	79	no	HBV	3.4	B	TACE	TACE	1,894	yes	1	1
**9**	PBMC+IHL	F	57	no	cryptogenic	2.7	B	resection+ TACE	TACE	71	no	1	1
**10**	PBMC	M	73	yes	NASH	2.4	A	RFTA	TACE	840	no	1	1
**11**	PBMC	M	69	yes	ethanol	6,999	B	none	n/a	n/a	n/a	0	0
**12**	PBMC	M	78	yes	HCV	4.6	A	PEI	RFTA	177	no	0	0

**13**	PBMC	M	68	yes	ethanol	2,166	A	RFTA+ TACE	TACE	44	yes	0	0
**14**	PBMC+TIL	M	74	yes	NASH	223.8	B	TACE	TACE	104	yes	2	2
**15**	PBMC	M	62	yes	ethanol	2,850	A	TACE	TACE	58	no	2	2
**16**	PBMC	M	75	yes	HCV	5.8	B	none	TACE	164	no	0	0
**17**	PBMC	M	79	no	cryptogenic	2.2	A	resection	RFTA+ TACE	729	no	0	0
**18**	IHL+TIL	M	56	yes	ethanol	7.6	A	none	resection	715	yes	3	2
**19**	PBMC+TIL	M	55	yes	ethanol	37.4	B	none	TACE	225	no	0	0
**20**	PBMC+TIL	M	57	yes	HBV	2.3	C	none	TACE	34	yes	4	2
**21**	PBMC	M	71	yes	cryptogenic	1.9	0	RFTA+ TACE	TACE	184	yes	2	1
**22**	PBMC	M	51	yes	ethanol	5.4	A	TACE	n/a	n/a	n/a	1	1
**23**	PBMC	M	72	yes	ethanol	8.9	0	none	resection	1,207	no	3	3
**24**	PBMC+IHL	M	53	yes	HCV	60,500	C	none	sorafenib	85	no	0	0
**25**	PBMC	M	62	yes	ethanol	1,051	C	none	resection	140	no	3	1
**26**	PBMC	M	82	yes	HCV	6.4	C	none	n/a	n/a	n/a	0	0
**27**	PBMC	M	69	yes	ethanol	889.0	C	TACE	TACE	124	no	1	1
**28**	PBMC+TIL	M	71	no	HCV	n/a	0	none	resection	n/a	n/a	1	1
**29**	PBMC	F	79	yes	HCV	9.2	A	none	TACE	218	no	0	0
**30**	PBMC +IHL+TIL	F	59	yes	HCV	441.2	0	TACE	resection	692	yes	6	4
**31**	PBMC	F	50	no	porphyria	5.2	A	none	resection	535	yes	0	0
**32**	PBMC	M	67	no	ethanol	2.2	0	none	resection	995	yes	2	2
**33**	PBMC	F	66	yes	ethanol	6.9	B	resection	RFTA	177	yes	0	0
**34**	PBMC+TIL	M	73	yes	cryptogenic	7.1	B	none	TACE	14	yes	1	1
**35**	PBMC+TIL	M	56	yes	HBV	119.3	C	none	TACE	22	no	0	0
**36**	PBMC	M	73	yes	HCV	65.0	0	TACE	TACE	288	no	3	3
**37**	PBMC +IHL+TIL	M	62	yes	cryptogenic	10.1	B	TACE	resection	776	yes	2	1

**38**	PBMC +IHL+TIL	M	68	yes	ethanol	n/a	B	n/a	resection	30	yes	1	1
**39**	PBMC	M	58	yes	cryptogenic	3.7	A	none	n/a	n/a	n/a	2	2
**40**	PBMC	M	62	no	HBV	16.7	A	TACE	TACE	729	no	1	1
**41**	PBMC	M	66	no	NASH	503.1	B	resection+ TACE	TACE	41	no	1	1
**42**	PBMC	M	63	yes	HCV	34.6	B	none	TACE	190	no	0	0
**43**	PBMC	M	79	yes	ethanol	3.7	C	RFTA+ TACE	TACE	282	no	0	0
**44**	PBMC+TIL	M	77	yes	ethanol	61.7	C	none	TACE	197	no	5	2
**45**	PBMC	F	82	yes	HBV	9.8	0	TACE	TACE	273	yes	7	4
**46**	PBMC+TIL	M	60	yes	HBV	2.2	A	none	TACE	71	no	2	1
**47**	PBMC	M	68	yes	ethanol	23.2	0	resection+ TACE	TACE	266	no	4	3
**48**	PBMC +IHL+TIL	F	73	no	cryptogenic	1,860	0	none	resection	274	no	4	2
**49**	PBMC	F	62	yes	AIH	598.8	B	none	TACE	419	no	1	1
**50**	PBMC	M	80	no	NASH	3.4	A	none	resection	146	yes	6	4
**51**	PBMC +IHL+TIL	M	61	no	cryptogenic	50.7	A	none	n/a	n/a	n/a	0	0
**52**	PBMC	M	69	yes	ethanol	7.1	B	resection+ TACE	TACE	145	no	0	0
**53**	PBMC	M	69	yes	ethanol	553.1	B	resection+ TACE	TACE	30	no	0	0
**54**	PBMC	M	65	yes	ethanol	135.6	C	n/a	n/a	n/a	n/a	0	0
**55**	PBMC	M	71	yes	ethanol	5.2	B	none	TACE	74	yes	3	3
**56**	PBMC	M	62	yes	NASH	7.9	A	resection	TACE	534	no	0	0
**57**	PBMC	M	66	yes	ethanol	285.0	A	none	TACE	92	no	3	2
**58**	PBMC	M	64	no	HCV	80.1	A	none	TACE	321	no	0	0
**59**	PBMC	M	63	yes	ethanol	56.4	D	none	n/a	n/a	n/a	1	1
**60**	PBMC	M	66	yes	HCV	106.2	B	none	TACE	135	no	0	0

**61**	PBMC+IHL	M	82	no	cryptogenic	3.3	0	none	resection	312	yes	2	2
**62**	PBMC	M	73	yes	HCV	24.5	A	none	TACE	485	yes	1	1
**63**	PBMC	M	81	no	NASH	3.8	B	none	TACE	394	yes	0	0
**64**	PBMC	F	45	yes	HBV	12,100	D	none	n/a	n/a	n/a	2	2
**65**	PBMC	M	55	yes	HCV	26,537	C	none	n/a	n/a	n/a	0	0
**66**	PBMC	M	73	yes	hemo-chromatosis	5.1	B	TACE	resection	212	no	0	0
**67**	PBMC+TIL	M	49	yes	HBV	8.6	B	none	resection	84	no	0	0
**68**	PBMC	M	82	no	HCV	4.0	B	none	TACE	92	no	0	0
**69**	PBMC	M	66	yes	ethanol	254.4	A	TACE	resection	93	no	0	0
**70**	PBMC	M	70	yes	ethanol	3.1	B	none	TACE	97	no	3	3
**71**	PBMC	M	59	yes	ethanol	60,500	D	none	n/a	n/a	n/a	0	0
**72**	PBMC	M	55	no	HCV	8.7	B	n/a	n/a	n/a	n/a	0	0
**73**	PBMC+TIL	M	57	no	cryptogenic	6,549	C	none	sorafenib	105	no	0	0
**74**	PBMC+IHL	M	85	no	HCV	1.4	A	none	TACE	110	no	2	2
**75**	PBMC	M	76	n/a	ethanol	6.0	A	n/a	resection	64	yes	1	1
**76**	PBMC	M	58	yes	HBV	54.1	A	none	resection	182	no	0	0
**77**	PBMC	M	77	yes	cryptogenic	12.9	B	TACE	TACE	28	no	0	0
**78**	PBMC	M	64	yes	HCV	30.3	B	resection	TACE	123	no	2	2
**79**	PBMC	M	58	yes	ethanol	375.2	A	none	TACE	393	yes	1	1
**80**	PBMC	M	81	yes	HCV	162.1	C	TACE+ sorafenib	TACE	44	no	0	0
**81**	PBMC	F	58	yes	HCV	3.9	0	none	n/a	n/a	n/a	0	0
**82**	PBMC	M	76	no	cryptogenic	4.0	B	none	n/a	n/a	n/a	0	0
**83**	PBMC+TIL	M	59	yes	ethanol	4.6	B	resection	resection	287	yes	0	0
**84**	PBMC	F	72	n/a	NASH	52.4	B	none	resection	303	yes	0	0
**85**	PBMC	M	58	yes	HCV	8.7	B	none	TACE	113	no	0	0
**86**	PBMC	M	76	no	cryptogenic	n/a	B	none	n/a	n/a	n/a	1	1
**87**	PBMC	M	83	yes	cryptogenic	55.4	B	TACE	TACE	279	no	8	4
**88**	PBMC	F	63	yes	PBC	4,703	B	TACE	TACE	84	yes	1	1

**89**	PBMC	M	75	no	ethanol	7.0	A	none	resection	227	yes	0	0
**90**	PBMC	M	72	yes	cryptogenic	60,500	A	resection	TACE	55	no	1	1
**91**	PBMC	M	73	yes	cryptogenic	41.2	B	none	TACE	63	no	0	0
**92**	PBMC	M	73	no	NASH	2.9	B	none	resection	143	no	0	0
**93**	PBMC	M	55	yes	ethanol	7,430	B	none	n/a	n/a	n/a	0	0
**94**	PBMC+IHL	n/a	n/a	no	HCV	n/a	n/a	n/a	n/a	n/a	n/a	1	1
**95**	PBMC	M	55	yes	HCV	155.0	C	none	TACE	16	no	0	0
**96**	PBMC	F	73	yes	cryptogenic	5.9	0	none	n/a	n/a	n/a	2	2

Supplementary Table 3 Known and newly fine-mapped CD8$^+$ T-cell epitopes found in the overlapping peptides recognized in the patient cohort.

Left: overlapping peptides and previously described epitopes contained within them. Right: frequent HLA-alleles in patients with responses to overlapping peptides and newly fine mapped epitopes. Epitope candidates were tested on antigen-unspecifically expanded CD8$^+$ T-cells of HLA-matched patients. nd: not done.

peptide	# of patients	previously described epitope	position	restricting HLA-allele (% of patients)	enriched HLA-alleles (% of patients)	newly fine-mapped epitope	position	responses (patients tested)
**AFP 1**	1	MKWVESIFL	1-9	A*02 (100)	-	-	-	-
**AFP 6**	17	-	-	-	A*02 (41)	-	-	-
					A*03 (41)	-	-	-
					B*07 (24)	-	-	-
					B*18 (24)	-	-	-
**AFP 52**	5	RSCGLFQKL	414-422	A*24 (20)	A*03 (40)	-	-	-
					B*08 (40)	-	-	-
**AFP 68**	4	GVALQTMKQ	542-550	A*02 (75)	A*03 (50)	-	-	-
					B*07 (50)	-	-	-
**GPC-3 36**	13	-	-	-	A*02 (31)	VMQGCMAGV	281-289	1 (37)
					A*03 (38)	-	-	-
					A*25 (15)	-	-	-
					B*44 (38)	-	-	-
**GPC-3 37**	1	EYILSLEEL	298-306	A*24 (0)	-	-	-	-
**GPC-3 65**	13	FLAELAYDL	522-530	A*02 (31)	A*03 (56)	QLRFLAELAY	519-528	2 (18)
					B*44 (38)	-	-	-
					B*51 (25)	-	-	-

**MAGE-A1 12**	6	SLFRAVITK	96-102	A*03 (50)	A*02 (50)	-	-	-
		ESLFRAVITK	95-102	A*11 (0)	B*07 (33)	-	-	-
					B*08 (33)	-	-	-
					B*44 (33)	-	-	-
**MAGE-A1 13**	1	ITKKVADLVGF	102-112	B*57 (0)	-	-	-	-
		KVADLVGFLL	105-114	A*02 (100)	-	-	-	-
**MAGE-A1 14**	8	KVADLVGFLL	105-114	A*02 (50)	A*01 (38)	-	-	-
					A*02 (38)	-	-	-
					A*03 (50)	-	-	-
					A*26 (25)	-	-	-
					B*38 (25)	-	-	-
**NY-ESO-1 11**	1	RLLEFYLAM	86-98	A*02 (100)	-	-	-	-
		LEFYLAMPF	88-98	B*18 (100)	-	-	-	-
**NY-ESO-1 12**	1	LAMPFATPM	92-100	B*35 (0)	-	-	-	-
				B*51 (0)	-	-	-	-
				Cw*03 (nd)	-	-	-	-
**NY-ESO-1 18**	6	-	-	-	A*02 (67)	LQLSISSCL	145-153	10 (37)
					A*66 (33)	-	-	-
					B*15 (50)	-	-	-
**NY-ESO-1 20**	12	SLLMWITQC	157-165	A*02 (58)	A*03 (33)	-	-	-
		SLLMWITQCF	157-166	A*02 (58)	B*07 (33)	-	-	-
		LLMQITQCF	158-166	A*02 (58)	-	-	-	-
		ITQCFLPVF	162-170	A*24 (0)	-	-	-	-
